# Quality identification of *Amomi fructus* using E-nose, HS-GC-IMS, and intelligent data fusion methods

**DOI:** 10.3389/fchem.2025.1544743

**Published:** 2025-02-06

**Authors:** Pan-Pan Zhang, Xin-Jing Gui, Xue-Hua Fan, Han-Li, Hai-Yang Li, Xiao-Peng Li, Feng-Yu Dong, Yan-Li Wang, Jing-Yao, Jun-Han Shi, Rui-Xin Liu

**Affiliations:** ^1^ School of Pharmacy, Henan University of Chinese Medicine, Zhengzhou, China; ^2^ Department of Pharmacy, The First Affiliated Hospital of Henan University of Chinese Medicine, Zhengzhou, China; ^3^ Henan Province Engineering Research Center for Clinical Application, Evaluation and Transformation of Traditional Chinese Medicine, Zhengzhou, China; ^4^ Henan Provincial Key Laboratory for Clinical Pharmacy of Traditional Chinese Medicine, Zhengzhou, China; ^5^ Beijing University of Chinese Medicine, Beijing, China; ^6^ Co-Construction Collaborative Innovation Center for Chinese Medicine and Respiratory Diseases by Henan and Education Ministry of China, Henan University of Chinese Medicine, Zhengzhou, China

**Keywords:** *Amomi fructus*, EN, HS-GC-IMS, intelligent data fusion, quality identification

## Abstract

*Amomi fructus* (AF) has been used for both medicinal and food purposes for centuries. However, issues such as source mixing, substandard quality, and product adulteration often affect its efficacy. This study used E-nose (EN) and headspace-gas chromatography-ion mobility spectrometry (HS-GC-IMS) to determine and analyze the volatile organic compounds (VOCs) in AF and its counterfeit products. A total of 111 VOCs were detected by HS-GC-IMS, with 101 tentatively identified. Orthogonal Partial Least Squares-Discriminant Analysis (OPLS-DA) identified 47 VOCs as differential markers for distinguishing authentic AF from counterfeits (VIP value >1 and *P* < 0.05). Based on the E-nose sensor response value and the peak volumes of the 111 VOCs, the unguided Principal Component Analysis (PCA), guided Principal Component Analysis-Discriminant Analysis (PCA-DA), and Partial Least Squares-Discriminant Analysis (PLS-DA) models were established to differentiate AF by authenticity, origin, and provenance. The authenticity identification model achieved 100.00% accuracy after PCA analysis, while the origin identification model and the provenance identification model were 95.65% (HS-GC-IMS: PLS-DA) and 98.18% (HS-GC-IMS: PCA-DA/PLS-DA), respectively. Further data-level fusion of E-nose and HS-GC-IMS significantly improved the accuracy of the origin identification model to 97.96% (PLS-DA), outperforming single-source data modeling. In conclusion, the intelligent data fusion algorithm based on E-nose and HS-GC-IMS data effectively identifies the authenticity, origin, and provenance of AF, providing a rapid and accurate method for quality evaluation.

## 1 Introduction

*AF* is one of the “Four major Southern Medicines” in China, derived from the dried and ripe fruit of *Amomum villosum* Lour, *A. villosum* Lour. var.*xanthioides* T.L.Wu et Senjen, or.

*Amomum longiligulare* T.L.Wu. First documented in the Theory of Medicinal Properties, it has been a commonly used traditional Chinese medicine in the past dynasties (Gui et al., 2022). AF is native to Yangchun, Guangdong Province, China, and is distributed in Guangdong Province, Yunnan, Guangxi, Hainan, and other places in China. It is produced in regions such as Myanmar and Vietnam. Among all the producing areas, Yangchun City in Guangdong and Yunnan Province are the most renowned (Yu et al., 2023). Known for its high quality, AF is regarded as an authentic southern medicine (Yin et al., 2024). It contains volatile oils, phenols, flavonoids, and polysaccharides, and possesses various pharmacological properties such as antioxidant, anti-infection, gastrointestinal mobility-promoting, anti-inflammatory, analgesic, antidiarrheal, and blood viscosity-reducing effects (Kim et al., 2020; Song et al., 2022). Functional digestive disorders, gastritis, acute lung injuries, *Helicobacter pylori* infection, and other conditions have been successfully treated with it (Suo et al., 2018; Zhao et al., 2022).

The cultivation of AF is challenging due to its unique floral structure, high requirements for growth, and low natural survival rates. As a result, the supply of AF decoction pieces is limited, leading to high market prices and significant variations in quality depending on origin. There are frequent issues with mixed sources and adulteration with similar products (Hou et al., 2023; Hou et al., 2019), such as, *A. longiligulare* T.L.Wu and *A. villosum* Lour. var. *xanthioides* T.L.Wu et Senjen as substitutes for high-quality AF (Hu et al., 2021), or mixing it with the related plant *Alpiniae oxyphyllae fructus* (Wang L. et al., 2021). This not only hinders standardization in clinical use but may also affect its therapeutic efficacy. Furthermore, due to the market value of AF and its superior stomach digestion and antidiarrheal properties compared to *A. longiligulare* and *A. villosum* Lour. var. *xanthioides* (Li et al., 2018), differences in chemical composition and efficacy have become a major focus of research.

Traditional identification of AF relies on visual, olfactory, taste, and tactile methods, which, although quick, require expertise and are highly subjective, making them unsuitable for routine, standardized application.

Advances in modern analytical technology and sensory science have led to the development of rapid detection methods for the quality evaluation of AF. These include authenticity identification based on visual fluorescent probes, this method quickly distinguishes the authenticity of AF through specific fluorescent labeling, and is both intuitive and efficient (Guo et al., 2021), the combination of high-performance liquid chromatography and chemometrics provides a more refined basis for distinguishing the authenticity of AF through complex chemical composition analysis (Doh et al., 2020), DNA barcoding analysis technology is based on the genetic information of AF, and it achieves authenticity identification by comparing specific DNA sequences (Doh et al., 2019) and gas chromatography fingerprint technology can also provide strong support for authenticity identification through the analysis of the volatile oil components in AF (Ding et al., 2004). Other techniques include ultra-high performance liquid chromatography-tandem quadrupole time-of-flight mass spectrometry technology enables the detection of multiple chemical components in AF with high sensitivity and high resolution, allowing for the tracing of its geographical origin through differences in these chemical components (Liu et al., 2017), electrochemical fingerprint technology, on the other hand, detects active components in AF. using electrochemical methods, with electrochemical signals serving as the basis for geographical origin identification (Wang et al., 2020a), and X-ray technology utilizes differences in the internal structure of AF to achieve rapid identification of its geographical origin through X-ray diffraction patterns (Wu et al., 2023). These methods, while accurate and reliable, require complex sample preparation, are time-consuming, expensive, and demand technical expertise, making them unsuitable for routine application.

Given that AF is rich in volatile components, the analysis of these compounds has become crucial for quality evaluation (Wang et al., 2016; Yu et al., 2018). Emerging bionic olfactory and flavor analysis technologies have also played a role in its evaluation. The electronic nose (E-nose), a bionic instrument simulating biological olfaction, can rapidly detect odor molecules by converting them into electrical signals. Due to its high sensitivity, reliability, and repeatability, it is widely used in identifying traditional Chinese medicines (Feng et al., 2021; Li et al., 2024a; Zhang et al., 2023a), origin identification (Feng et al., 2022; Gan et al., 2024), quality evaluation (Jing et al., 2022; Li et al., 2024b) and food freshness detection (Xu et al., 2024).

HS-GC-IMS is a newer technology that uses the drift time of ions in an electric field for material analysis. It combines gas chromatography with ion mobility spectrometry for secondary separation, offering high resolution, sensitivity, and non-destructive, rapid analysis (Tong et al., 2019). A wide range of traditional Chinese medicines have been evaluated using this method (Dai et al., 2024; Zhang et al., 2023b), food classification, and flavor characterization (Feng et al., 2022; Xiang et al., 2023). However, there are few reports on the application of E-nose and HS-GC-IMS to the quality evaluation of AF.

Data fusion technology, initially developed by the U.S. Navy for military applications (Li et al., 2023; Wang et al., 2021a), has been applied to food (Jandric et al., 2015) and medicine (Li et al., 2024a; Li et al., 2024b). This technology integrates multiple complementary data sources, leveraging their synergy to provide more detailed and accurate characterization than single-source data (Borras et al., 2015).

This study aims to evaluate the quality of AF using an intelligent data fusion method combining E-nose and HS-GC-IMS. First, the E-nose response values of AF and its counterfeits were analyzed. Second, the VOCs of AF and its counterfeits were characterized using HS-GC-IMS, and potential markers were identified. Next, PCA-DA and PLS-DA models were constructed to identify authenticity, origin, and provenance of AF based on single-source data from E-nose and HS-GC-IMS respectively. Finally, data-level fusion of E-nose and HS-GC-IMS data was used to improve the accuracy of origin and provenance identification. In this paper, we establish a rapid and accurate method for evaluating the quality of AF as a base for evaluating the quality of other foods and drugs.

## 2 Experimental materials and methods

### 2.1 Samples and reagents

In this study, 55 batches of AF were collected from five key producing areas. This included 21 batches from Yunnan, China (S1-S21), 13 batches from Guangdong, China (S22-S34), 11 batches from Guangxi, China (S35-S45), 5 batches from Hainan, China (S46-S50), and 5 batches from Myanmar (S51-S55). The Yunnan and Guangdong samples were identified (AF-1:S1-S34), while the samples from Guangxi and Myanmar were identified as *A. villosum* Lour. var.*xanthioides* T.L.Wu et Senjen (AF-2:S35-S45, S51-S55). The Hainan samples were classified as *A. longiligulare* T.L.Wu (AF-3: S46-S50) (Hou et al., 2023).

**FIGURE 1:**
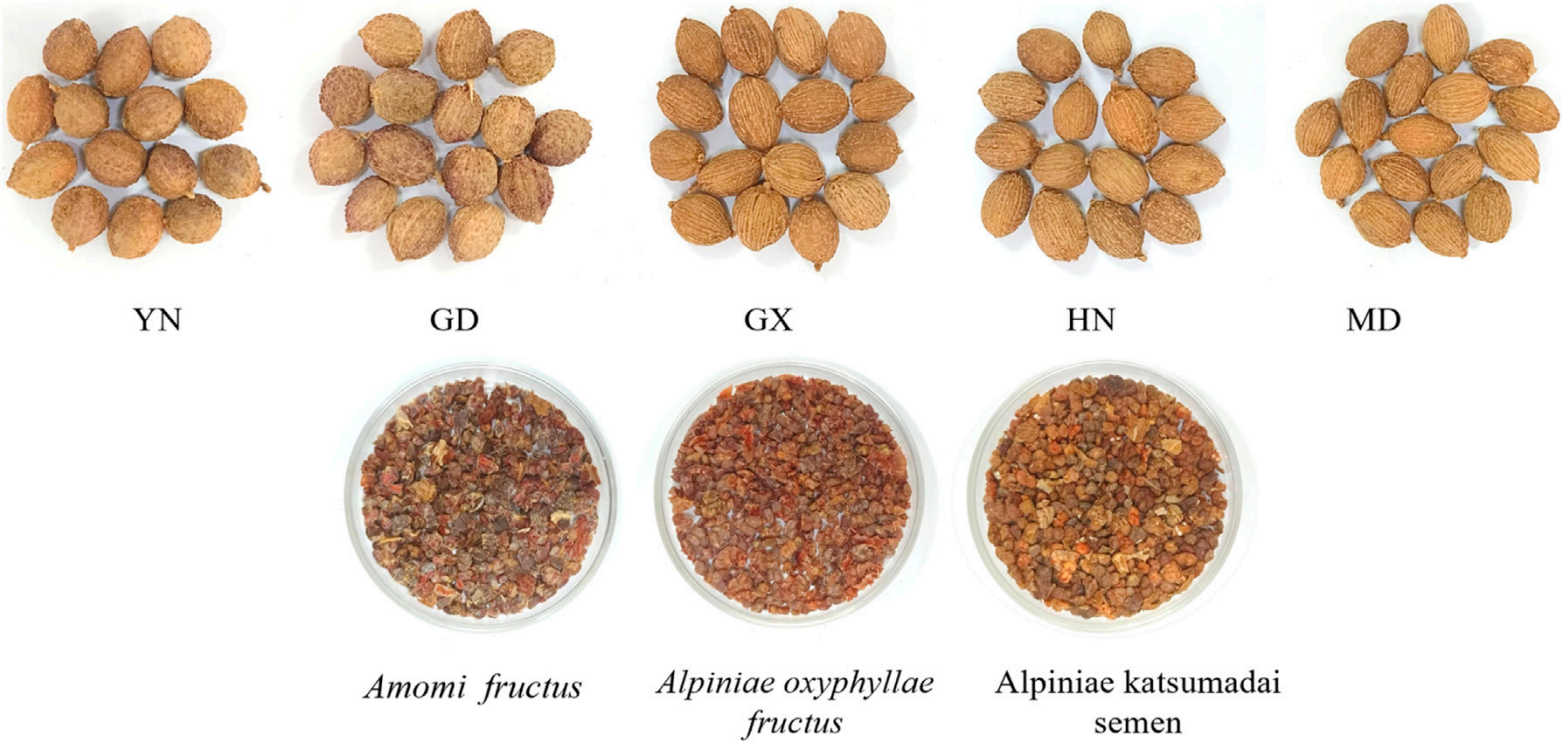
*Amomi fructus* (AF) from different origins and its counterfeits.

In addition, 10 batches each of *Alpiniae oxyphyllae fructus* and *Alpiniae katsumadai semen* were collected as counterfeits for comparison. Among them, *Alpinia oxyphylla Miq* is AO (S56-S65) and *Alpinia katsumadai* Hayata is AK (S66-S75) (Figure 1). All 75 batches were pulverized and sieved through the No.3 sieve (The average diameter of the sieve: 355 μm ± 13 μm; Number of mesh: 50) of Chinese Pharmacopoeia for E-nose detection. For HS-GC-IMS analysis, the samples were coarsely ground. Sample information is in Supplementary Table 1.

The reagents used included chromatographically pure n-alkanes (2-butanone, 2-pentanone, 2-hexanone, 2-heptanone, 2-octanone, and 2-nonanone), purchased from Shanghai Aladdin Biochemical Technology Co., Ltd, Shanghai, China. Other analytically pure reagents were sourced from Tianjin Hengxing Chemical reagent manufacturing Co., LTD, Tianjin, China.

### 2.2 E-nose analysis

Using the electronic nose PEN3 (AirSENSE, Germany), 10 metal oxide sensors were used to collect E-nose data (W1C, W5S, W3C, W6S, W5C, W1S, W1W, W2S, W2W, W3S) (Supplementary Table 2). Compound levels can be determined by response values obtained by each sensor for different types of chemicals.

For the analysis, Parallel precision weighing S1-S75 sample powder 3.0 g 3 parts, placed in a 50 mL headspace bottle, standing for 30 min, to be tested. The injection needle was inserted into the sealed sample cup containing the sample by direct headspace suction method for detection. The PEN3 device was then connected to a computer, with data collection software running. The sample inlet flow rate was set to 400 mL min^-1^, the preparation time was 5 s, the sampling time was 100 s, the sensor balance time was 100 s, and the data when each injection time was 80 s were taken as the balance point data. The total measurement time of each sample is about 3 min and the average value of three parallel tests is obtained.

### 2.3 HS-GC-IMS analysis

A Flavour Spec^®^ flavor analyzer (GAS, Germany), equipped with analytical software such as Laboratory Analytical Viewer (LAV), Reporter, Gallery Plot, Dynamic PCA plug-ins, and the GC IMS Library Search software, was used for HS-GC-IMS analysis. Each sample (0.1 g) was precisely weighed and placed in a 20 mL headspace bottle.

The headspace injection conditions were set to an incubation temperature of 80°C, an incubation time of 15 min, an incubation speed of 500 rpm, and an injection needle temperature of 85°C; and injection volume, 0.1 mL. Each sample was injected 3 times in parallel.

For gas chromatography, an MXT-WAX strong polar chromatographic column (15 m × 0.53 mm, 1.0 μm, Restek, United States of America) was employed. The column temperature was set to 80°C, and the carrier gas was high-purity nitrogen (≥99.999%). The carrier gas flow rate was programmed as follows: 2 mL. min^-1^ for 2 min, 2–10 mL. min^-1^ for 8 min, 10–100 mL. min^-1^ for 10 min, 100 mL. min^-1^ for 10 min.

### 2.4 Data analysis

For HS-GC-IMS, n-alkanes served as external references to calculate the retention index (RI). The built-in NIST and IMS databases were used to characterize the VOCs.

Using Reporter, Gallery Plot, and other plug-ins, topographic maps and VOC fingerprints were generated and for sample analysis. Peak volume data for each sample were analyzed using orthogonal partial least squares discriminant analysis (OPLS-DA) with SIMCA 14.1 software to identify the differential markers.

OPLS-DA is a regression modeling method for multiple dependent and independent variables, removing noise unrelated to classification. It highlights classification-relevant principal components and improves simplicity, analytical ability, and effectiveness of the model, providing better insight into group differences and predictions for sample grouping (Xiao et al., 2022).

### 2.5 Data-level fusion

Data-level fusion (Li et al., 2024c) directly combines the two types of detection information for each sample (EN and HS-GC-IMS). Only one fusion method is used.

### 2.6 Model construction method

The authenticity, origin, and provenance identification models for samples were established using Matlab R2022 software (MathWorks, United States of America). The peak volume data (or two-source fusion information) of the volatile components from HS-GC-IMS were used as the independent variable matrix X. In the determination of the benchmark information Y, the authenticity identification model used the values 1, 2, and 3 to represent AF, AO, and AK, respectively. For origin identification, the values 1, 2, 3, 4, and 5 represented YN, GD, GX, HN, and MD, respectively. In the provenance identification model, 1, 2, and 3 represented AF-1, AF-2, and AF-3, respectively.

The classification 6.0 toolkit, built into the software, was used to evaluate model performance via leave-one-out cross validation. PCA-DA and PLS-DA models (Hou et al., 2023) were established, and the prediction accuracy of each model was calculated. These two methods, guided analytical statistical methods (Chen et al., 2024), aided in the discovery of relevant sample-information within the data set. PCA was performed using SIMCA 14.1, a commonly used unsupervised stoichiometry tool that simplifies multidimensional variables into a linear combination of a few original variables.

## 3 Experimental results discussion

### 3.1 E-nose results

**FIGURE 2:**
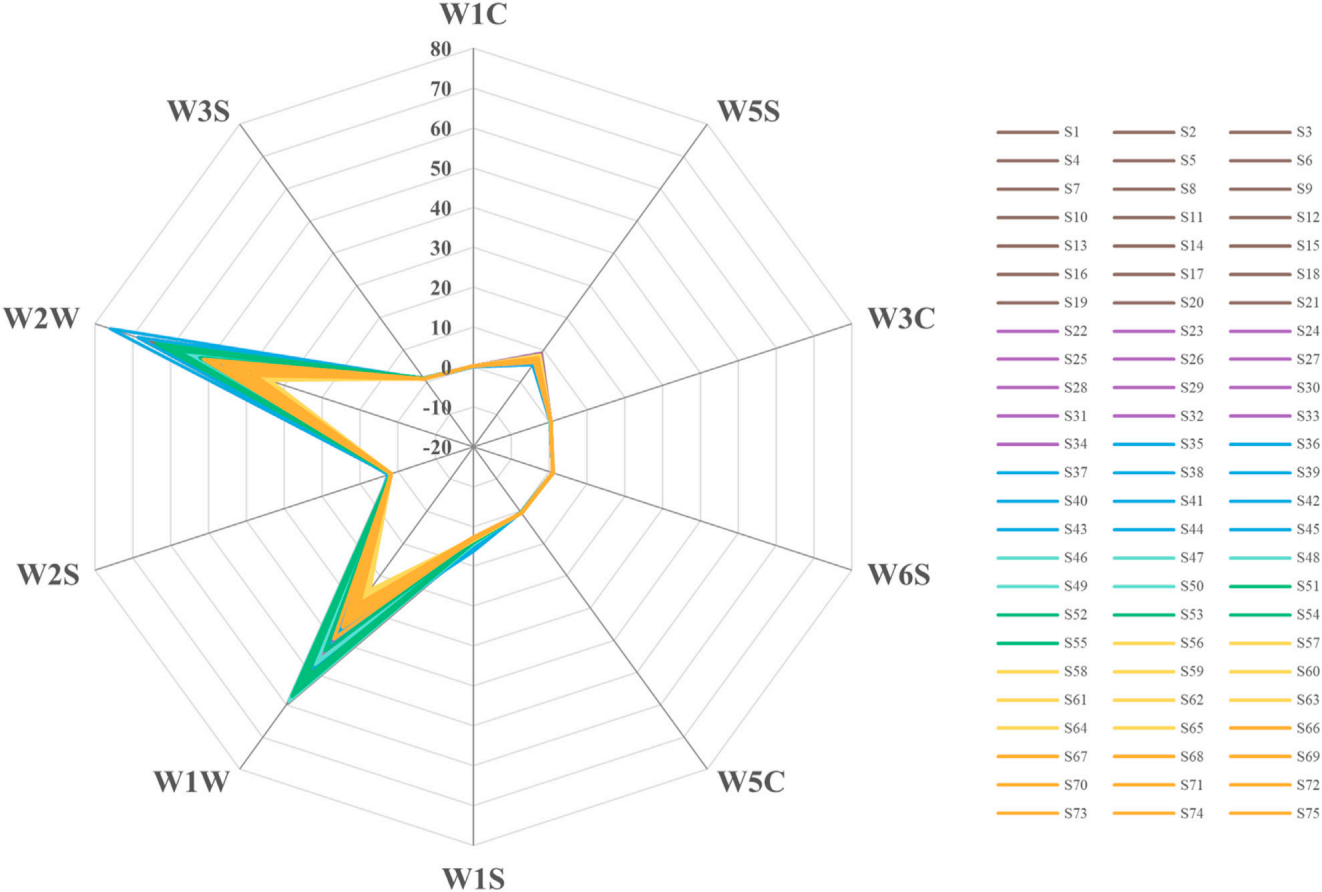
Electronic nose output information radar map.

Ten bionic olfactory response values were obtained from the measured samples. All sensor response values were greater than 0. Most samples demonstrated higher response values on W1W and W2W, followed by W5S (Figure 2).

The Wilk’s Lambda value of the variation information carried by the 10 electronic nose sensors is shown in Supplementary Figure 1A. The Wilk’s Lambda histogram reflects the variation information value carried by each sensor. The smaller the value, the more the variation information the variable carries. From Supplementary Figure 1B, sensors No. 1, No. 3, No. 5, and No. Nine carried more variable information, contributing significantly to the model. Sensor No. Four carried the least variable information and had the smallest contribution to the classification of AF and its counterfeits.

In combination with Supplementary Figure 1B, it is evident that sensor No. Nine was a positively correlated variable, while sensors No. 1, No. 3, and No. Five were negatively correlated variables. Sensor No. Four was near the latent variable load diagram’s circle point, indicating minimal contribution to sample classification. The compound information represented by No.1, No.3, No.5 and No.9 sensors is shown in Supplementary Table 2.

Based on the previously published quality evaluation method of AF using gas phase, E-tongue, and E-nose (Hou et al., 2023), PCA-DA and PLS-DA identification models were established to determine the authenticity, origin, and provenance of AF decoction pieces. The authenticity identification model achieved a 100.00% accuracy rate, while the origin identification model performed poorly, with a maximum accuracy rate of 87.00% (Table 1). The PCA, PLS-DA, and PCA-DA model scores are shown in Supplementary Figure 2. These results show the need for further improvements. Therefore, the focus shifts toward developing a fusion modeling method for origin and provenance identification to enhance data utilization and model performance.

**TABLE 1:** Classification and accuracy of each model.

Technology	Category	Model	Cross-validation accuracy (percent)	Misassignation	Not assigned
E-nose	authenticity	PCA-DA	100.00	0	0
		PLS-DA	100.00	0	S21,S26,S75
	origin	PCA-DA	78.18	S1,S2,S21,S24,S25,S26,S30,S47,S48,S51,S54,S55	0
		PLS-DA	87.18	S2,S3,S5,S9,S51	S2,S16,S18,S19,S25,S35,S37,S41,S46,S47,S48,S50,S52,S53,S54,S55
	provenance	PCA-DA	90.91	S21,S51,S53,S54,S55	0
		PLS-DA	95.74	S46,S51	S21,S35,S47,S48,S52,S53,S54,S55
HS-GC-IMS	authenticity	PCA-DA	100.00	0	0
		PLS-DA	100.00	0	S68
	origin	PCA-DA	90.91	S42,S47,S49,S50,S51	0
		PLS-DA	95.65	S42,S49	S11,S15,S40,S41,S47,S48,S50,S51,S55
	provenance	PCA-DA	98.18	S49	0
		PLS-DA	98.18	S49	0
E-nose + HS-GC-IMS (Dataset fusion)	origin	PCA-DA	90.91	S35,S49,S50,S51,S54	0
		PLS-DA	97.96	S48,S49	S11,S35,S40,S41,S50,S51
	provenance	PCA-DA	94.55	S49,S51,S54	0
		PLS-DA	96.23	S49	S11,S48,S50,S51

### 3.2 HS-GC-IMS results

#### 3.2.1 Preliminary comparison of HS-GC-IMS spectra and qualitative analysis of VOCs

**FIGURE 3:**
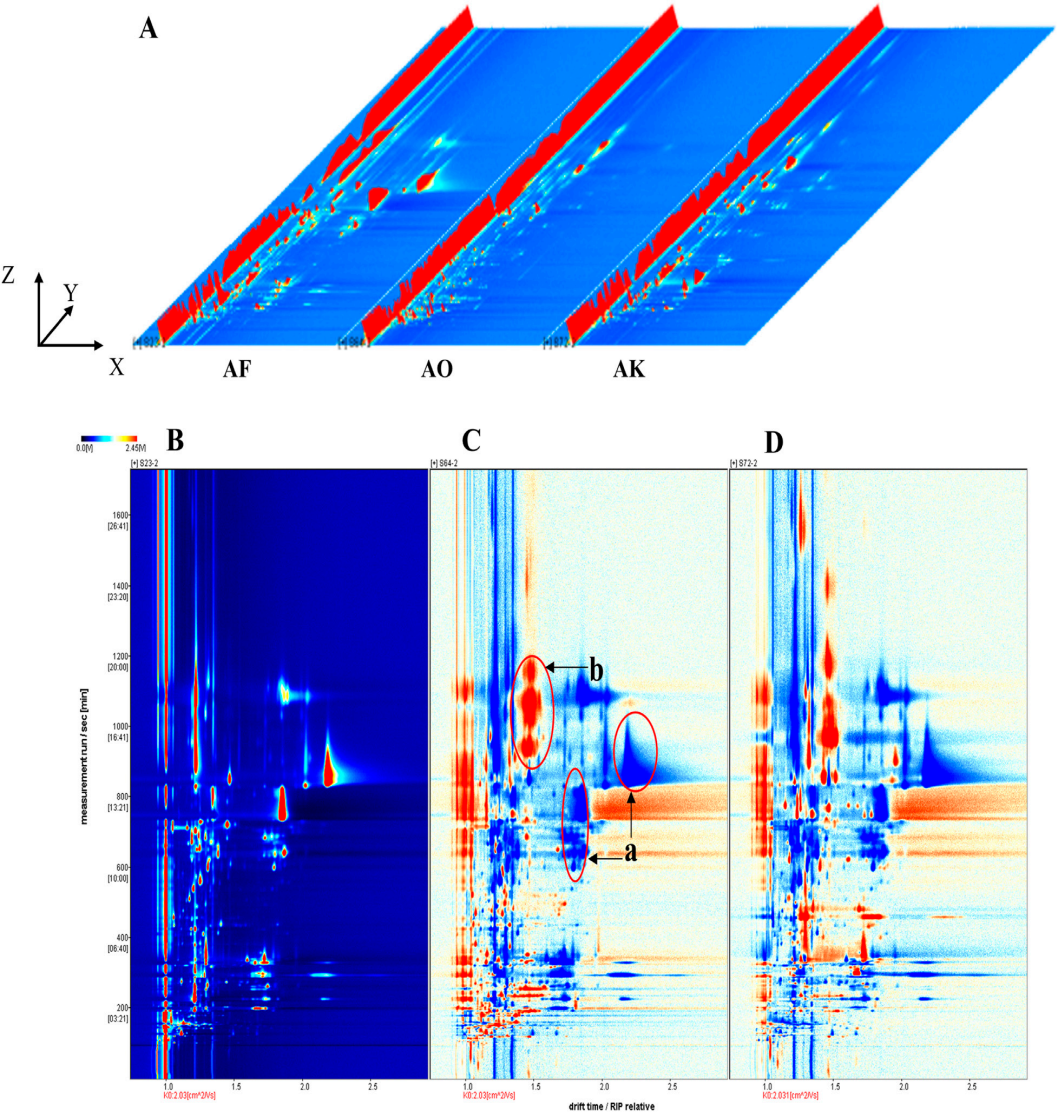
Three-dimensional spectra of volatile compounds in AF and its counterfeits, gas-phase ion migration spectra, and different diagrams of samples. The *X*, *Y* and *Z*-axes represent migration time, retention time, and peak intensity, respectively. **(A)** three-dimensional spectra of AF and its counterfeits. **(B)** two-dimensional gas phase ion migration spectrum of AF. **(C, D)** comparative difference spectra of *Alpiniae oxyphyllae fructus* and *Alpiniae katsumadai semen* with AF as reference, respectively.

The volatile components in *Amomi fructus* and its counterfeits were analyzed by LAV software. To compare the VOC variations among the samples, we utilized both two-dimensional and three-dimensional spectra for a comprehensive analysis. The three-dimensional spatial distribution (Figure 3A) revealed significant differences in the types and signal intensities of volatile compounds between AF and its counterfeit products. In order to delve deeper into the distinctions, a bird’s eye view with two dimensions was employed for examination. In this unique perspective, the vertical axis symbolizes the retention time in gas chromatography, while the horizontal axis signifies the normalized ion migration time (normalized). The background is blue, with the red vertical line at abscissa 1.0 indicating the reactive ion peak (RIP). The RIP refers to the peak that forms on the mass spectrum, representing a specific reactive ion or compound ion, after the sample has undergone separation by gas chromatography and detection by ion migration spectrometry. Each dot on both sides of the RIP represents a compound. The redder the dot color, the larger the area, the more the content of the component; the bluer the dot color, the smaller the area, the less the content of the component (Li et al., 2024b).

Samples S23–2, S64-2, and S72-2 were randomly selected to compare the HS-GC-IMS top views of AF and its counterfeit decoction pieces (*Alpiniae oxyphyllae fructus* and *Alpiniae katsumadai semen*). The ion peaks were most evident at retention times between 100 and 1,600 s and drift times of 1.0–2.5 s (Figure 3B). AVOC difference comparison was performed by using the spectrum of S23-2 as the reference and subtracting the spectra of other samples (Figure 3B). White indicates that the VOCs of the two are consistent, red indicates higher VOC content compared to the reference (area b), and blue indicates lower content (area a). These significant differences in VOC content and type further confirm the distinct odors of AF and its counterfeits. Some red regions may serve as potential markers to distinguish between them, while the blue regions are indicative of the reference sample’s characteristic.

To qualitatively differentiate VOC types in AF and its counterfeits, the NIST and IMS databases were used for identification. In general, drift and retention times are allowed a deviation of ±5% from standard materials (Wang et al., 2020b). The qualitative results of the samples were shown in Supplementary Table 3 and Figure 3. A total of 111 VOCs were detected, with 55 in AF and 56 VOCs in the counterfeits, resulting in 101 tentatively identified VOCs. When selecting these 111 volatile components, our primary criteria were as follows: Firstly, these components exhibited strong volatility in the samples, making them easily detectable through methods such as gas chromatography. Secondly, they possessed stable chemical properties, enabling them to maintain good stability during the analysis process. Thirdly, these components were closely related to our research objectives and could provide us with crucial information about the sample characteristics. Lastly, we also took into account the detection range and accuracy of the instrumentation to ensure that the selected components could be accurately and reliably detected under our experimental conditions. These VOCs included 32 esters (28.83%), 13 ketones (11.71%), 12 alcohols (10.81%), and alkenes, phenols, pyrazines, aldehydes, ethers, acids, and other compounds (48.65%). Eleven were detected as monomers and dimers (e.g., 2-phenylethyl butyrate, ethyl 2-hydroxybenzoate, allyl phenoxyacetate, (E)-geraniol, 1-octanol, (E)-caryophyllene, longifolene, 4-methylguaiacol, 2-ethyl-3,5-dimethylpyrazine, 2-ethyl-5-methylpyrazine, and 2,2,4,6,6-pentamethylheptane). The presence of both monomers and dimers, as in the case of longifolene, is likely due to the high concentration and proton affinity of related compounds (Li et al., 2019).

#### 3.2.2 Overall comparative analysis of VOC fingerprints

**FIGURE 4:**
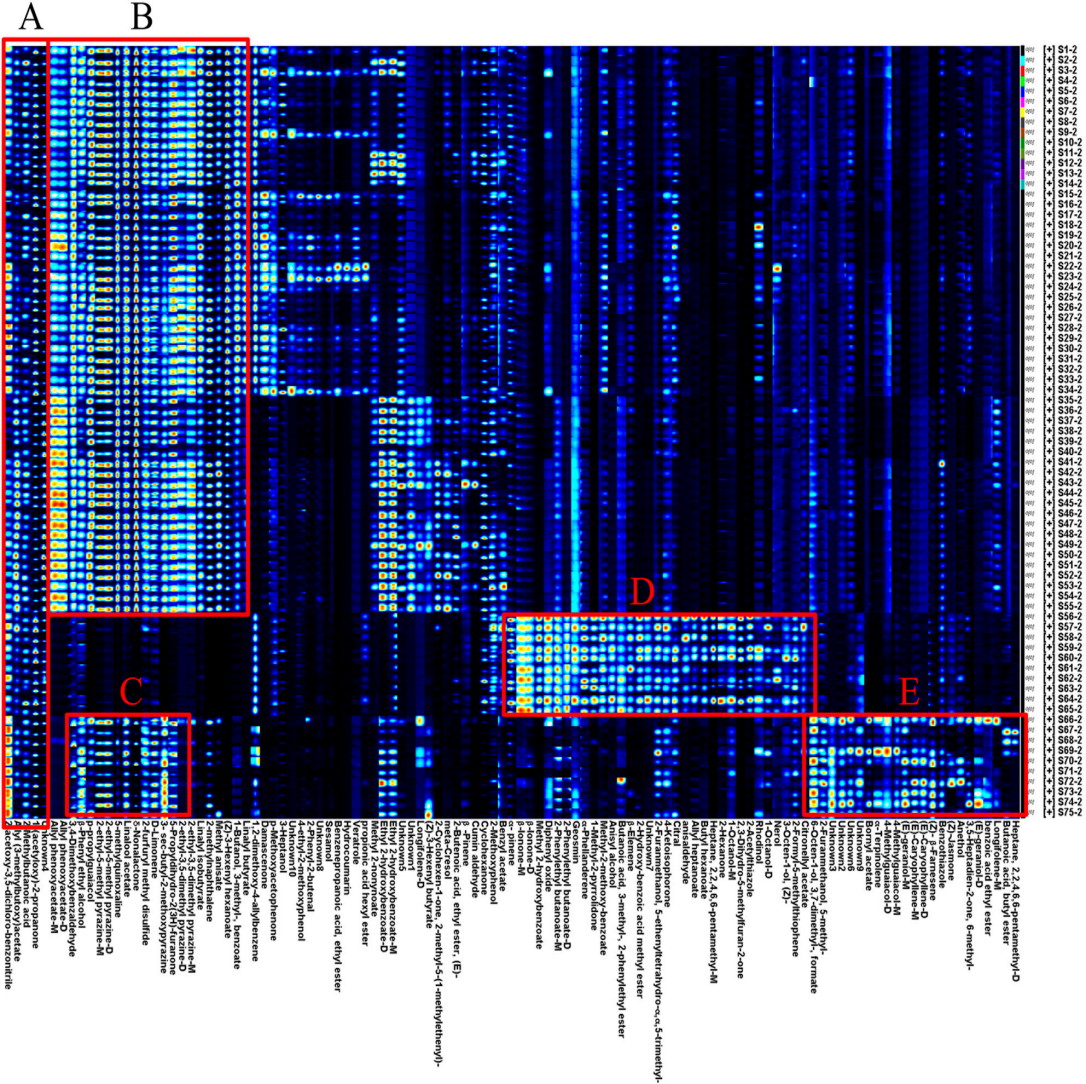
HS-GC-IMS fingerprint of volatile odor components of AF, *Alpiniae oxyphyllae fructus,* and *Alpiniae katsumadai semen* decoction pieces. From the region **(A-E)** indicates different kinds of volatile components.

Using the peak signal from the topographic map, the volatile fingerprints detected by HS-GC-IMS were analyzed and compared across samples (Figure 4). Each row represents the signal peaks from a single sample, while each column represents the same VOCs across different samples (Feng et al., 2022).

The analysis showed clear differences in VOCs between AF and its counterfeits. In region B, esters, alcohols, aldehydes, and pyrazines had higher peak response values in AF compared to *Alpiniae oxyphyllae fructus* and *Alpiniae katsumadai semen.* In region D, ketones, esters, alcohols, and alkenes had higher peak response values in *Alpiniae oxyphyllae fructus*, and compounds such as α-pinene and geosmin were identified as characteristic VOCs for *Alpiniae oxyphyllae fructus*. Similarly, in region E, esters, alcohols, alkenes, and phenols ahad higher peak response values in *Alpiniae katsumadai semen*, with compounds such as bornyl acetate and (E)-geraniol-M serving as characteristic VOCs for *Alpiniae katsumadai semen*.

However, five volatile components (2-acetoxy-3,5-dichloro-benzonitrile, allyl (3-methylbutoxy) acetate, 2-methylbutanoic acid, 1-(acetyloxy)-2-propanone, and Unknown4) were present in both AF and its counterfeit products were identified in region A. These shared components may contribute to the difficulty in distinguishing between AF and its counterfeits products.

#### 3.2.3 Screening of differential markers of volatile components in AF and its counterfeits

**FIGURE 5:**
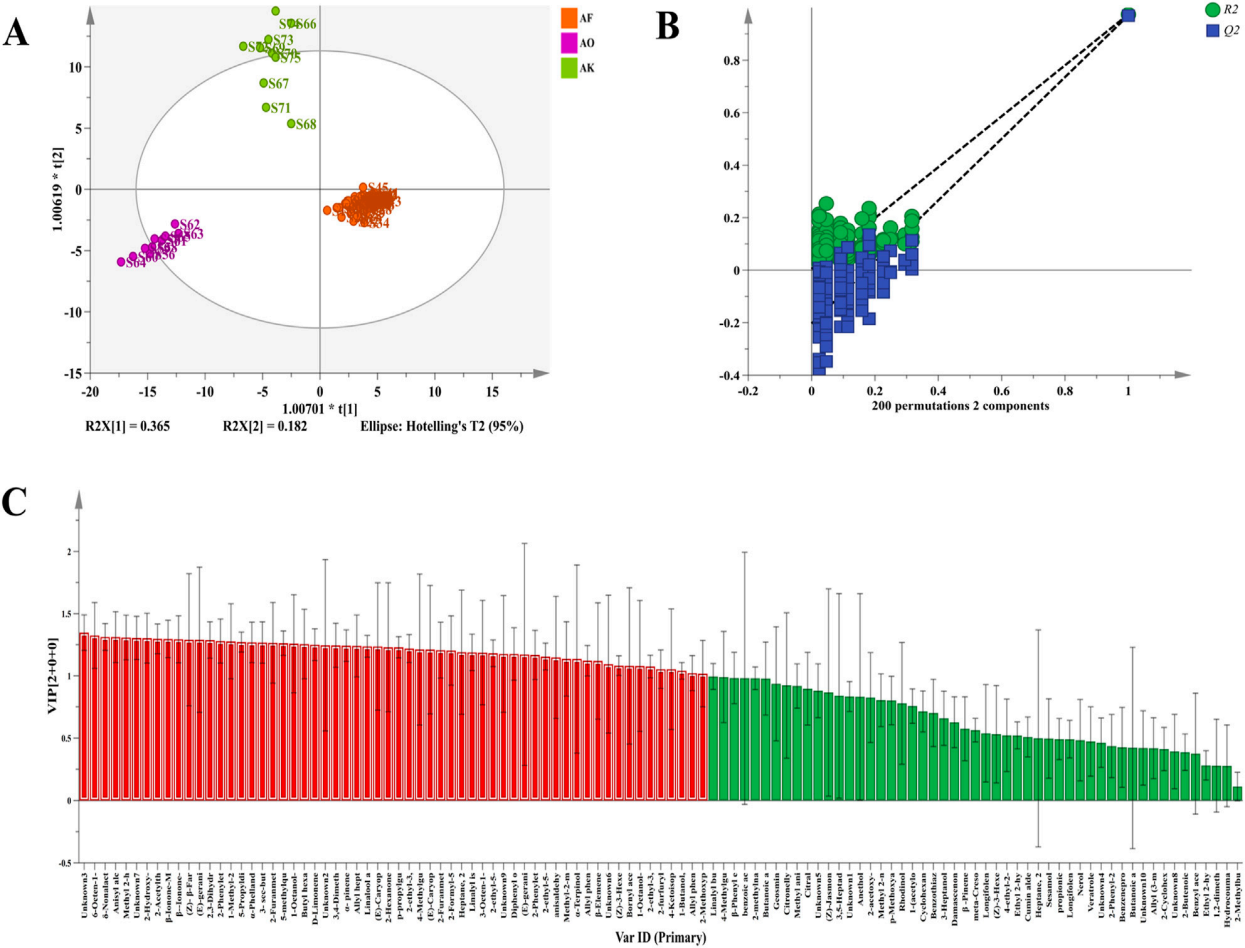
Multivariate statistical analysis of differential peaks of volatile components of AF, *Alpiniae oxyphyllae fructus,* and *Alpiniae katsumadai semen* decoction pieces. **(A)** OPLS-DA score chart. **(B)** OPLS-DA model permutation test diagram. **(C)** VIP value of the OPLS-DA model.

To further identify the differential markers of volatile components in AF and its counterfeits, the peak volume of 111 volatile components was used as the independent variable, while the authenticity information served as the dependent variable. An OPLS-DA analysis was performed using SIMCA 14.1 software (Xiao et al., 2022). The first two principal components were plotted into a two-dimensional score map, as shown in Figure 5A. The model’s performance, characterized by *R* ^*2*^ *Y* and *Q* ^*2,*^ was 0.959 and 0.952, respectively, demonstrating excellent classification accuracy. A permutation test of the OPLS-DA model (*n* = 200 times) confirmed that the *Q* ^*2*^ and *R* ^*2*^ values on the left side were lower than the original points on the right side (Feng et al., 2024). The *Q* ^*2*^ and *R* ^*2*^ randomly arranged at the left end are smaller than the original values at the right end, and the regression curve of *Q* ^*2*^ is compared with the negative axis of the Y-axis, and the *R* ^*2*^ values are all greater than 0 (Figure 5B), indicating that the model fits well and there is no over-fitting phenomenon. Thus, the 111 detected VOCs can serve as reference markers for distinguishing AF from *Alpiniae oxyphyllae fructus* and *Alpiniae katsumadai semen*.

The variable importance projection (VIP) from the OPLS-DA model is shown in Figure 5C. The higher the VIP value, the greater the group difference, making it crucial for discriminant classification (Scavarda et al., 2021). With a VIP value >1 and *p* < 0.05, 47 volatile components (Supplementary Table 4) were screened. Among these, 43 VOCs were qualitatively identified, comprising 12 esters, 6 alcohols, 6 alkenes, 5 ketones, 5 pyrazines and various phenols, aldehydes, ethers, dioxanes, acids, thiophenes, and thio files. These VOCs can thus be used as differential markers to distinguish AF from its counterfeits.

### 3.3 Study on the quality identification method of AF based on HS-GC-IMS information

#### 3.3.1 Authenticity identification

**FIGURE 6:**
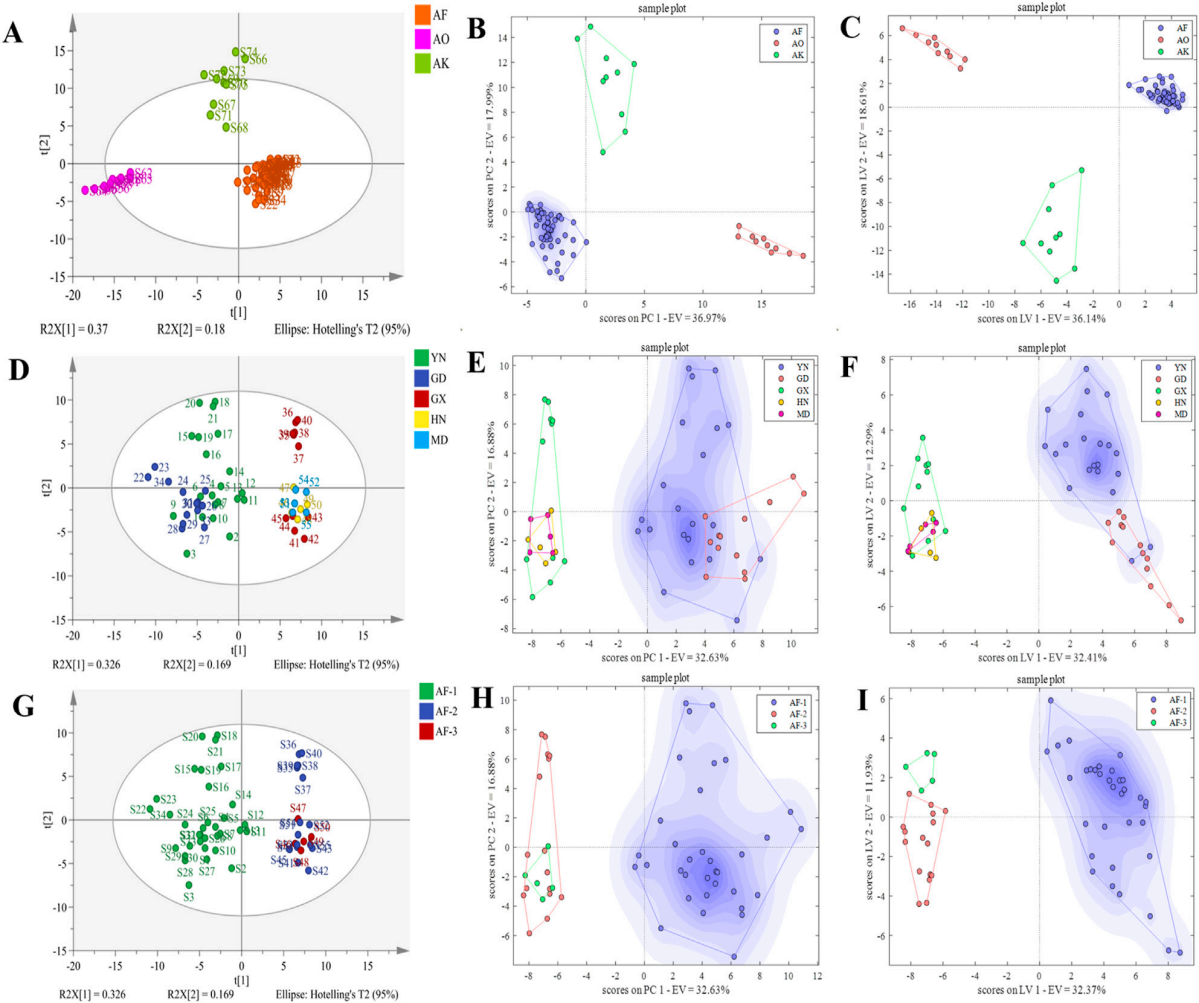
PCA, PCA-DA, and PLS-DA score plots for authenticity, origin, and provenance identification. **(A–C)** PCA, PCA-DA, and PLS-DA score plots for authenticity. **(D–F)** PCA, PCA-DA, and PLS-DA score plots for origin. **(G–I)** PCA, PCA-DA, and PLS-DA score plots for provenance.

HS-GC-IMS peak volume data from 55 batches of AF, 10 batches of AO*,* and 10 batches of AK were analyzed using PCA, PCA-DA, and PLS-DA. The PCA two-dimensional plot (Figure 6A) indicated clear clustering into distinct categories.

In the PCA-DA identification model (Figure 6B), selecting 20 principal components yielded the lowest classification rate error, explaining more than 96.00% of the sample variation (Supplementary Figure 4B). Cross-validation accuracy reached 100%, with no unclassified or misclassified samples. Similarly, in the PLS-DA identification model (Figure 6C), selecting 19 latent variables has the highest classification accuracy explaining 94.00% of the variation (Supplementary Figure 4D). Cross-validation accuracy was also 100% though sample S68 was unclassified, likely due to salt-processing differences. These results indicate that both models can effectively identify AF and its counterfeit slices.

#### 3.3.2 Origin identification

HS-GC-IMS peak volume data from 21 batches of AF from Yunnan, 13 from Guangdong, 11 from Guangxi, 5 from Hainan, and 5 from Myanmar were analyzed using PCA, PCA-DA, and PLS-DA. The PCA results (Figure 6D) indicated that the samples could not be clearly clustered by origin, implying that the volatile components are relatively similar across origins. Therefore, unsupervised PCA analysis alone could not effectively distinguish them.

In the PCA-DA identification model (Figure 6E), selecting 15 principal components resulted in the lowest classification error rate, explaining more than 89.00% of the sample variation (Supplementary Figure 5B). Cross-validation accuracy was 90.91%, with misclassifications from Guangxi, Hainan, and Myanmar, but no unclassified samples. The PLS-DA model (Figure 6F), explained 80.00% of the variation with 10 latent variables (Supplementary Figure 5D), achieving 95.65% cross-variation accuracy. However, there were misclassified and unclassified samples, indicating that the models’ performance in identifying AF origins requires further improvement.

#### 3.3.3 Provenance identification

HS-GC-IMS peak volume data from 34 batches of AF-1, 16 batches of AF-2, and 5 batches of AF-3 were analyzed using PCA, PCA-DA, and PLS-DA. The PCA results (Figure 6G) revealed that only AF could be clearly distinguished, as the other two samples shared similar flavor profiles. Like the origin analysis, unsupervised PCA alone could not effectively differentiate them.

In the PCA-DA identification model (Figure 6H), 20 principal components explained over 94.00% of the sample variation (Supplementary Figure 6B), achieving 98.18% cross-validation accuracy. Misclassification occurred with sample S49 of *A. longiligulare*, but no samples were unclassified. In the PLS-DA model (Figure 6I), 8 latent variables explained 74.00% of the variation (Supplementary Figure 6D), with the misclassification and unclassification results as the PCA-DA model. These results indicate that both models can effectively identify different provenance slices of AF.

### 3.4 Study on the quality identification method of AF based on HS-GC-IMS and E-Nose data fusion

The accuracy of the qualitative identification model for distinguishing genuine and fake Amomum based on single-source sensory information reached 100.00%, but the accuracy of the origin and provenance identification models was relatively low. To improve data utilization and model prediction accuracy, this section applies data-level fusion of the two types of detection information collected above. The fused data is used as the X value, and the classification information of the origin and provenance of Amomum samples is used as the Y value. PCA-DA and PLS-DA were employed to establish the origin and provenance identification models of AF, and the models were evaluated using leave-one-out cross-validation.

#### 3.4.1 Origin identification

**FIGURE 7:**
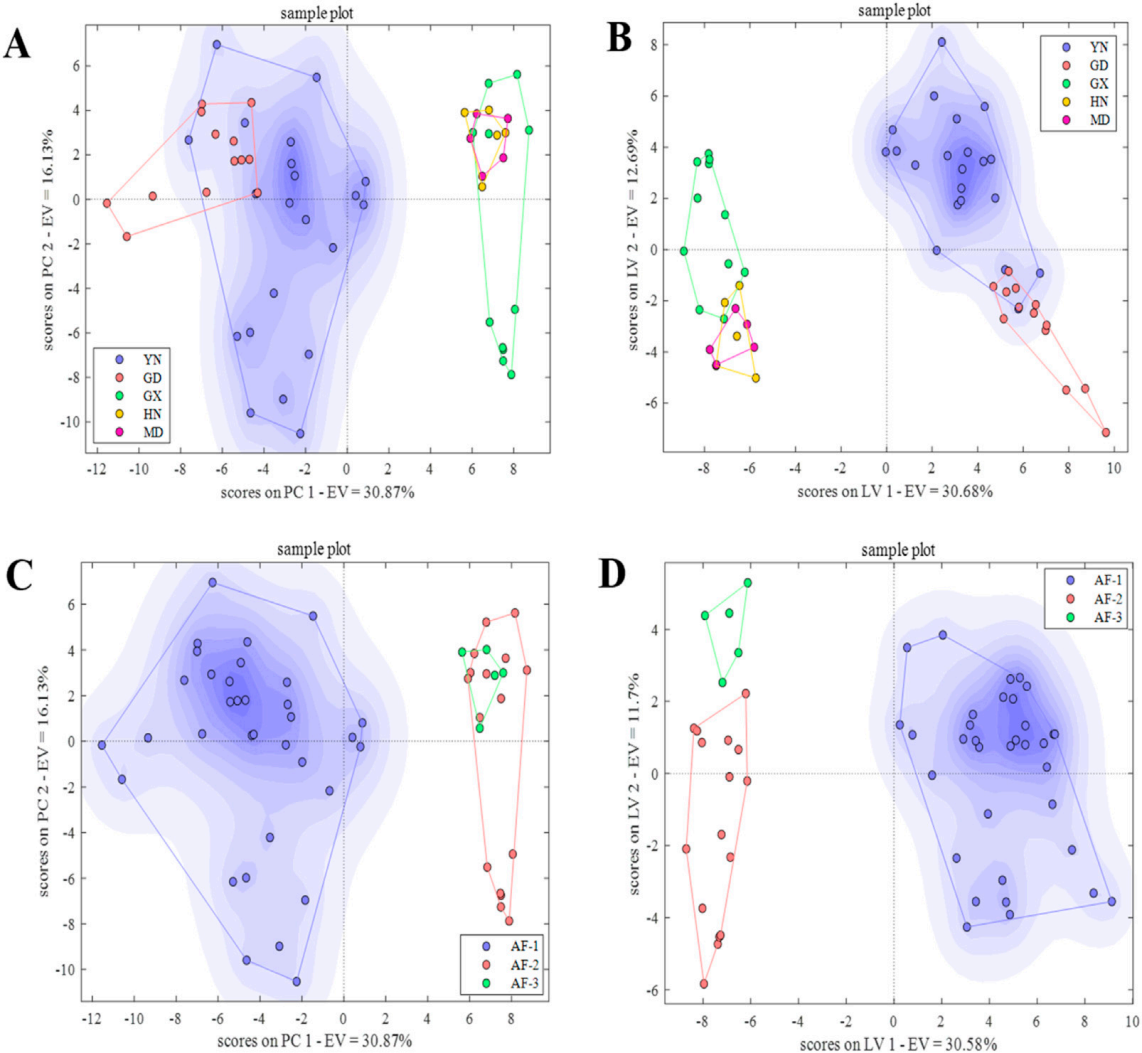
PCA-DA and PLS-DA score plots for origin and provenance identification based on data fusion. **(A, B)** PCA-DA and PLS-DA score plots for origin. **(C, D)** PCA-DA and PLS-DA score plots for provenance.

In the PCA-DA identification model (Figure 7A), when 20 principal components were selected, the model classification error rate was the lowest, and the first 20 principal components explained more than 93.00% of the sample variation (Supplementary Figure 7B). The model’s cross-validation accuracy was 90.91%, with 1, 3, and 1 misclassified samples from Guangxi, Hainan, and Myanmar, respectively, and no unclassified samples. The results were consistent with the single-source classification results. In the PLS-DA model (Figure 7B), using 09 latent variables, the model classification accuracy was highest, explaining 76.00% of the variation (Supplementary Figure 7D). The cross-validation accuracy was 97.96%, with only 1 misclassified sample from Guangxi and 6 unclassified samples from Yunnan, Guangxi, Hainan, and Myanmar. Compared to the single-source classification results, the number of misclassified and unclassified samples significantly decreased. Overall, the origin identification model of AF based on data fusion improved the accuracy of the PCA-DA and PLS-DA models by 12.13% and 10.18%, respectively, compared to the single-source (E-nose) (Figure 7B). The fused PLS-DA model performed better, improving accuracy by 2.31% compared to the optimal single-source (HS-GC-IMS) model (Table 1)., indicating that data fusion provided certain advantages in obtaining multi-level sample information, thereby improving the prediction accuracy.

#### 3.4.2 Provenance identification

In the PCA-DA identification model (Figure 7C), selecting 15 principal components resulted in the lowest classification error rate, with the first 15 principal components explaining more than 88.00% of the sample variation (Supplementary Figure 8B). The cross-validation accuracy was 94.55%, with 1 and 2 misclassified samples from AF-2 and AF-3, respectively. There were no unclassified samples. In the PLS-DA model (Figure 7D), using 19 latent variables, the classification accuracy was higher, explaining 89.00% of the variation (Supplementary Figure 8D). The cross-validation accuracy was 96.23%, with 2 misclassified samples from AF-2, and 1 unclassified sample each from AF-2 and AF-3. Overall, the accuracy of the PCA-DA and PLS-DA models for provenance identification improved by 3.64% and 0.49%, respectively, compared to the lower-accuracy single-source (E-nose) model (Table 1). Data fusion had advantages in obtaining multi-level information, improving the model’s prediction accuracy to a certain extent.

### 3.5 Discussion

#### 3.5.1 Distinct from previous research

In the earlier research (Hou et al., 2023), the authentication of AF was limited by a relatively small sample size, encompassing only 64 samples. Additionally, in terms of geographical origin identification, the study only included samples from four locations: Yunnan, Guangdong, Hainan in China, and Myanmar. However, in this study, we have made significant progress. We have introduced the advanced HS-GC-IMS identification technology, which has greatly enhanced the accuracy and efficiency of authenticating the genuineness of AF.

In terms of authenticity identification, the increase in sample size to 75 has enhanced the reliability and universality of the research results. In terms of geographical origin identification, this study has expanded upon the original four origins by adding samples from Guangxi, China, making the sources of origin more diverse and comprehensive. This expansion not only aids in our deeper understanding of the growth characteristics and quality differences of AF in different geographical environments but also provides richer data support for traceability and quality control of AF.

Furthermore, this study has, for the first time, effectively discriminated between the different provenances of AF (Ai et al., 2023). As a traditional Chinese medicinal material, the diversity of its provenances has a significant impact on the quality and pharmacological effects of the herb. Through meticulous identification work, we are able to more accurately distinguish between AF of different botanical origins, providing a more scientific basis for its rational use and quality control.

Meanwhile, scholars have utilized HS-GC-IMS technology to identify 30 volatile compounds in AF. Upon comparing these compounds with the 101 volatile compounds identified in this study, it was found that there were approximately 10 common components shared between the two. However, the primary focus of these scholars was on developing a novel multi-stage continuous combined drying technique, which involves hot air drying with humidity and temperature control followed by radio frequency drying. They conducted in-depth research on various quality aspects such as the optimal moisture transition points, drying efficiency, shell cracking rates, color, microstructure, volatile compounds, total flavonoid content, and antioxidant activity under three two-stage continuous combined drying methods. In contrast, this study utilized the compound information obtained through HS-GC-IMS analysis to effectively identify the authenticity, origin, and provenance of AF, achieving satisfactory identification results.

#### 3.5.2 Examine deficiencies and constraints of methods

Insufficient sample collection. During the quality evaluation and related research of AF, due to resource constraints, geographical distribution, and cooperation difficulties, the collected number of AF samples failed to meet expectations, thereby affecting the comprehensiveness and accuracy of the research. In the later stages, by increasing resource investment, broadening sample sources, optimizing sample collection methods, strengthening data sharing and cooperation, and adjusting research methods and objectives, we can gradually overcome the issue of insufficient sample collection and promote in-depth development of the quality evaluation and related research of AF.

The equipment requires regular maintenance. Although this method may demonstrate good stability and reliability in the short term, in the long run, it may encounter performance degradation due to factors such as equipment aging and environmental changes. Therefore, regular maintenance, calibration, and updating of model parameters are necessary to ensure the long-term stability of this method.

The algorithmic challenges of intelligent data fusion. Flexibility in algorithm design: Intelligent data fusion algorithms need to fully consider the complexity and diversity of volatile components in AF, as well as the variability between different batches of samples. Therefore, the design of these algorithms must possess a high degree of flexibility and adaptability to cope with various complex situations. Optimization of algorithm performance: After designing the algorithm, extensive experimental verification and performance optimization are required. This includes improving the recognition accuracy of the algorithm, reducing false positives and false negatives, and other key indicators, to ensure the accuracy and reliability of the evaluation results.

In summary, the method combining electronic noses, HS-GC-IMS, and intelligent data fusion holds broad application prospects in the field of AF quality evaluation. However, it also faces many challenges and limitations. In the future, we need to continue exploring and innovating to overcome these challenges and promote the continuous development and improvement of AF quality evaluation technology.

## 4 Conclusion

By combining E-nose, HS-GC-IMS technology, and intelligent data fusion methods, we can basically rapidly distinguish the authenticity, origin, and provenance of AF, ideal results have been achieved in terms of authenticity identification, and the model discrimination effect after data fusion is superior to that of modeling based on a single data source. This study not only helps to reveal the differences in flavor components between AF and its adulterants but also provides innovative ideas for quality assessment of other foods and medicines.

## Data Availability

The original contributions presented in the study are included in the article/Supplementary Material; further inquiries can be directed to the corresponding author.
