# Supplementary material for: Quality identification of Amomi fructus using E-nose, HS-GC-IMS, and intelligent data fusion methods
Source: Front Chem. 2025 Feb 6;13:1544743. doi: 10.3389/fchem.2025.1544743 (PMC11840568; doi:10.3389/fchem.2025.1544743)
Supplement: Supplementary file 1 [file DataSheet1.docx]

Supplementary Material

# Supplementary Figures and Tables

## Supplementary Figures

**
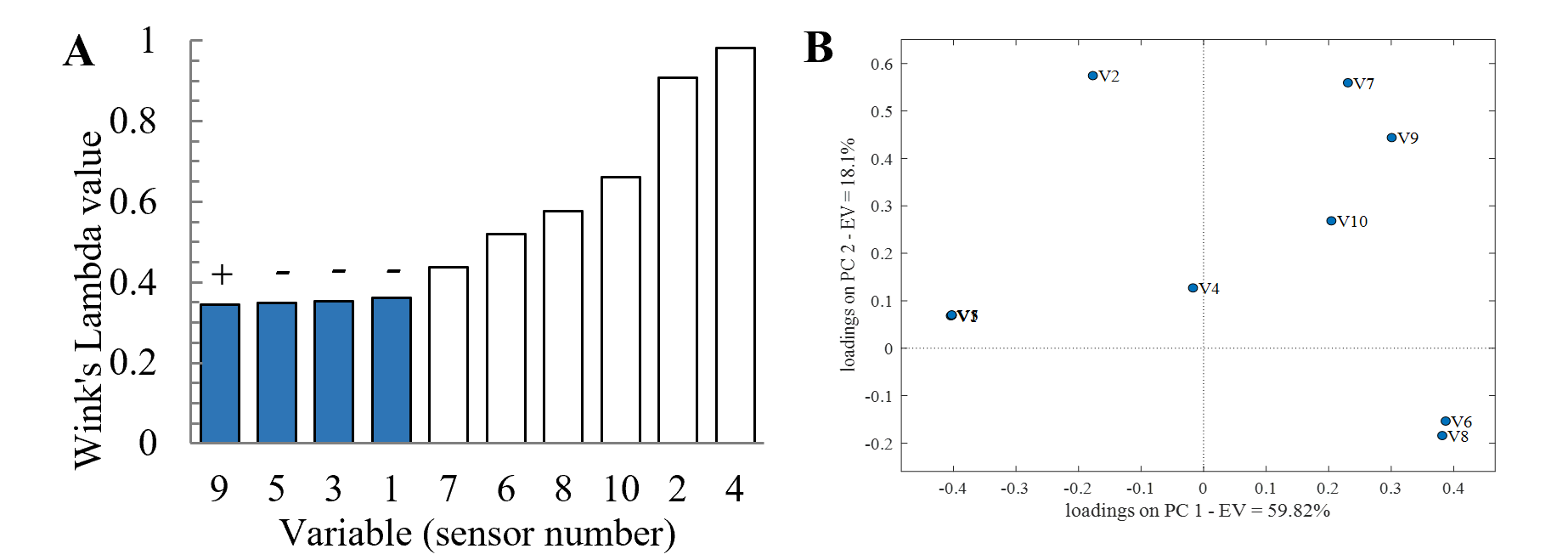
**

**Figure 1.** Variable contribution analysis of PCA-DA identification model based on electronic nose for *Amomi fructus* and its counterfeit products. **(A)** Wink 's lambda value histogram. **(B)** PCA-DA identification model load diagram


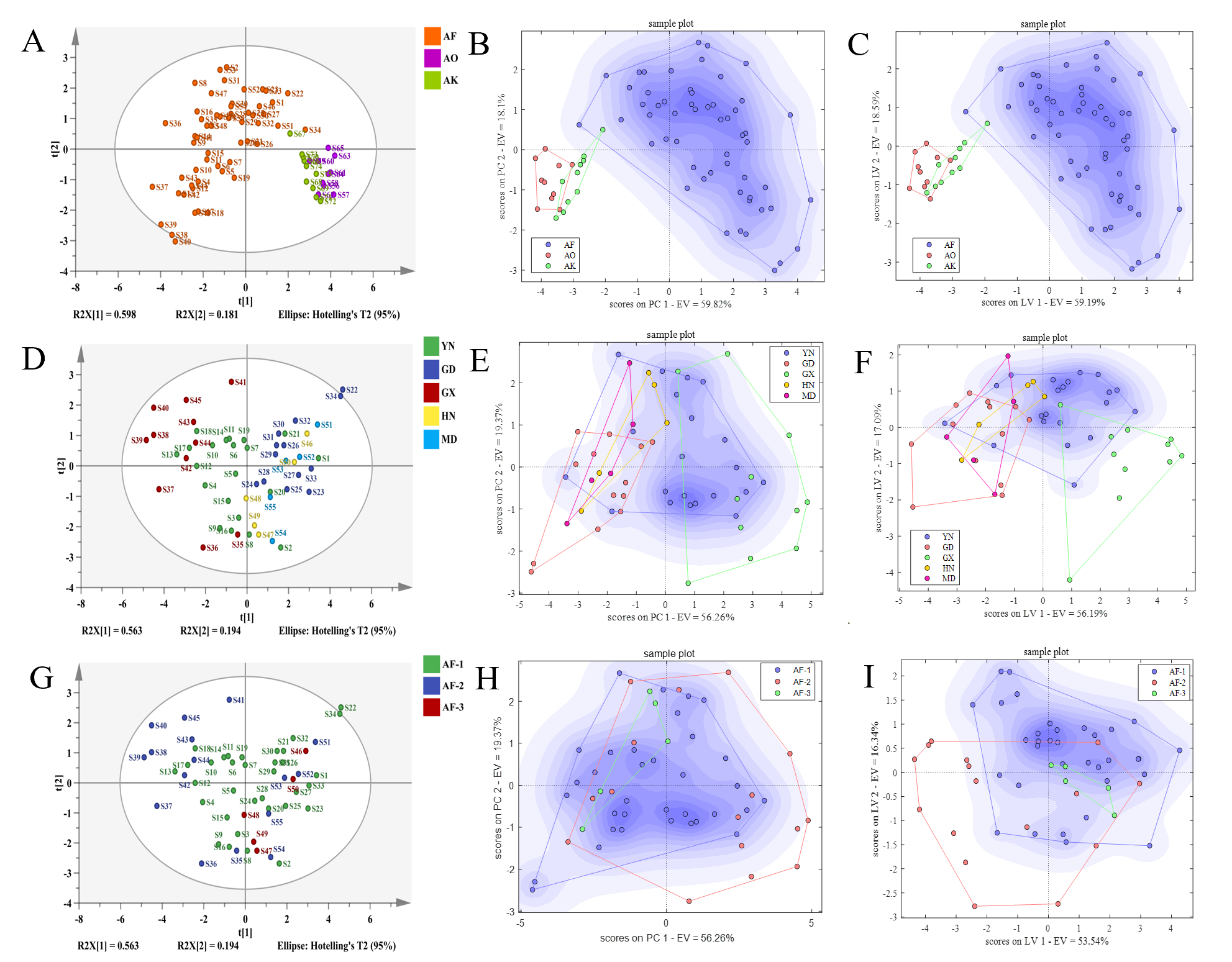


**Figure 2.** The model score diagram of electronic nose authenticity, origin and provenance identification. **(A, B, C)** authentic PCA, PCA-DA, PLS-DA. **(D, E, F)** origin PCA, PCA-DA, PLS-DA. **(G, H, I)** provenance PCA, PCA-DA, PLS-DA.

Table 4 Compound list information


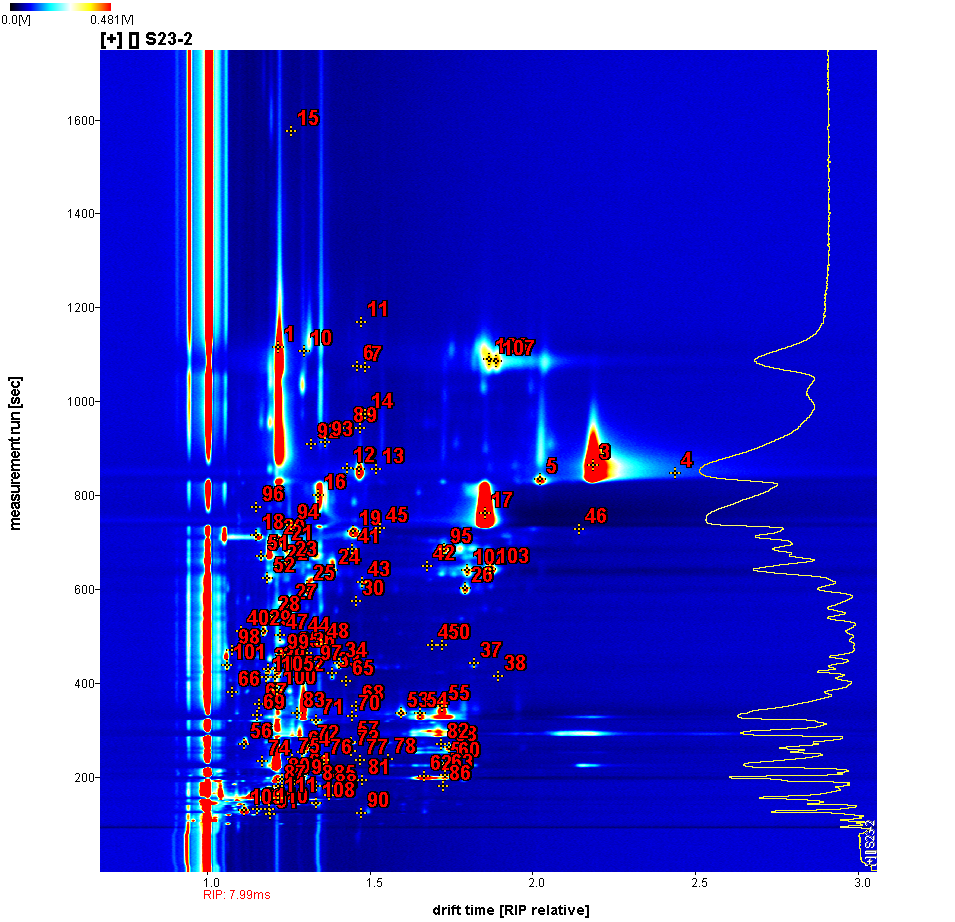


**Figure 3.** Point map of volatile components in *Amomi fructus* and its counterfeits


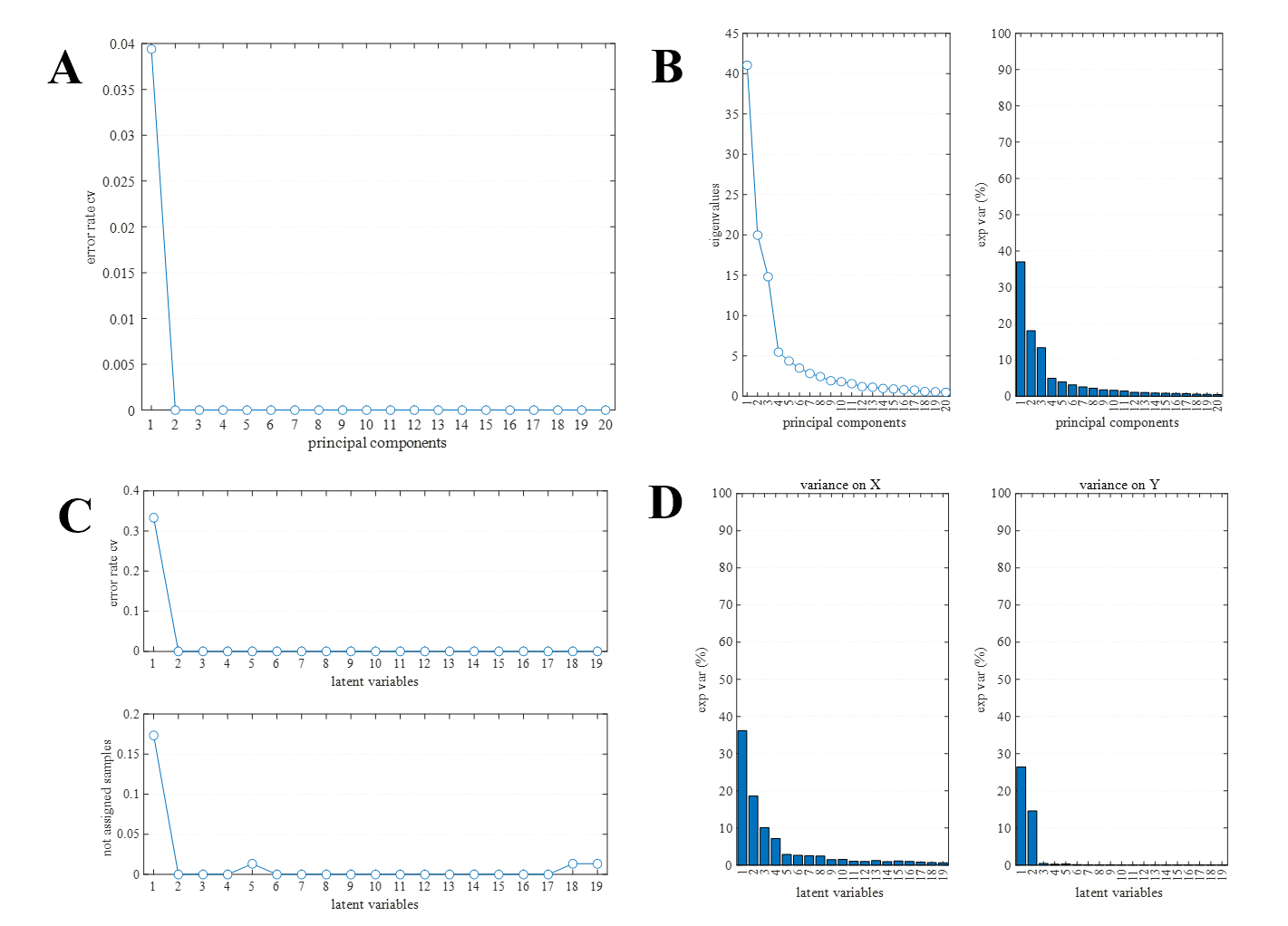


**Figure 4.** The cross validation error diagram and the explained variation diagram of the authenticity identification model of *Amomi fructus* based on HS-GC-IMS were established. **(A)** Principal component fraction-cross validation error plot in PCA-DA model. **(B)** Variation infographic of PCA-DA latent variable interpretation. **(C)** The number of latent variables-cross validation error plot in PLS-DA model. **(D)** Variation infographic of PLS-DA latent variable interpretation.


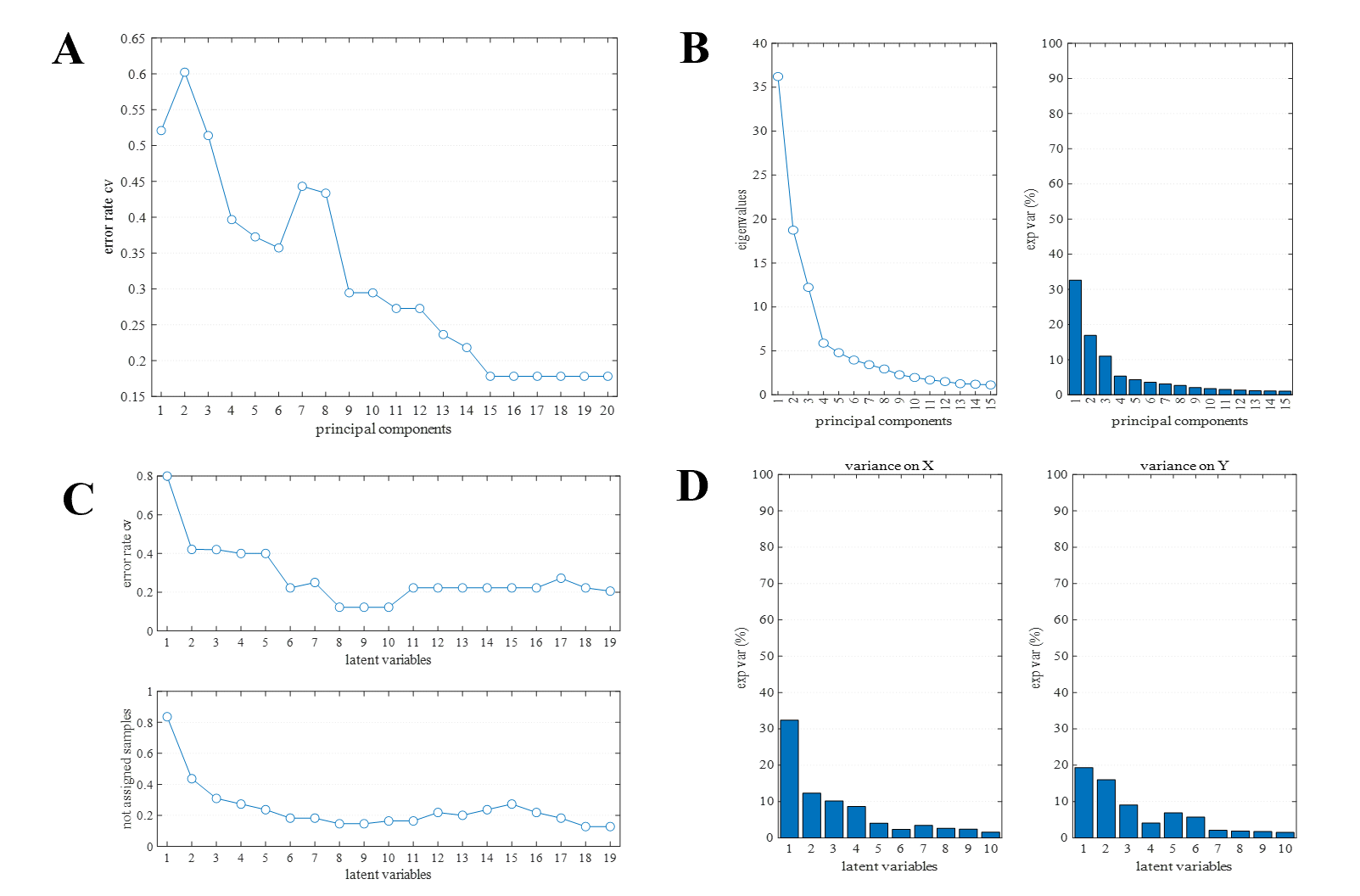


**Figure 5.** The cross validation error diagram and explained variation diagram of the origin identification model of *Amomi fructus* based on HS-GC-IMS were established. **(A)** Principal component fraction-cross validation error plot in PCA-DA model. **(B)** Variation infographic of PCA-DA latent variable interpretation. **(C)** The number of latent variables-cross validation error plot in PLS-DA model. **(D)** Variation infographic of PLS-DA latent variable interpretation.


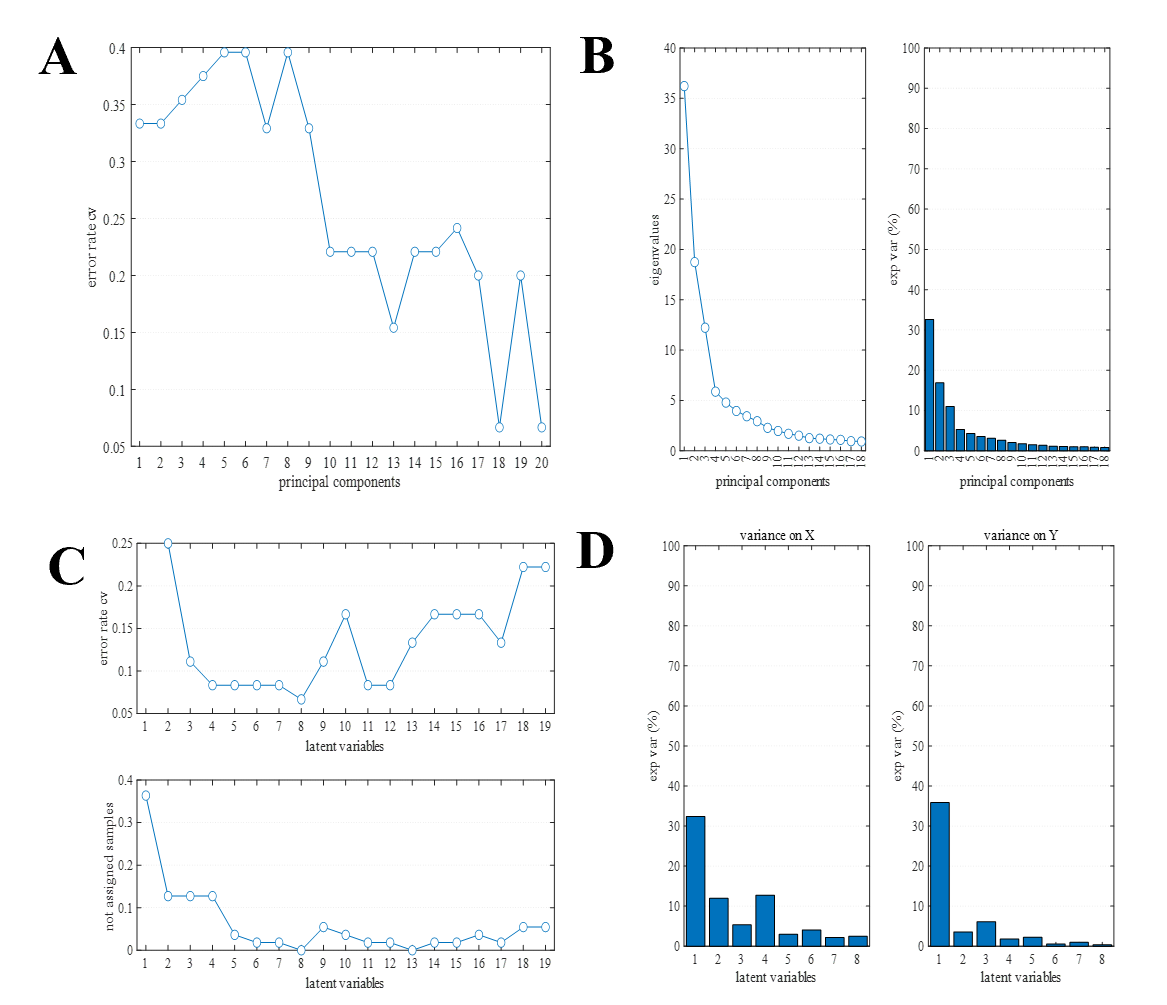


**Figure 6.** The cross validation error diagram and explained variation diagram of the provenance identification model of *Amomi fructus* based on HS-GC-IMS were established. **(A)** Principal component fraction-cross validation error plot in PCA-DA model. **(B)** Variation infographic of PCA-DA latent variable interpretation. **(C)** The number of latent variables-cross validation error plot in PLS-DA model. **(D)** Variation infographic of PLS-DA latent variable interpretation.


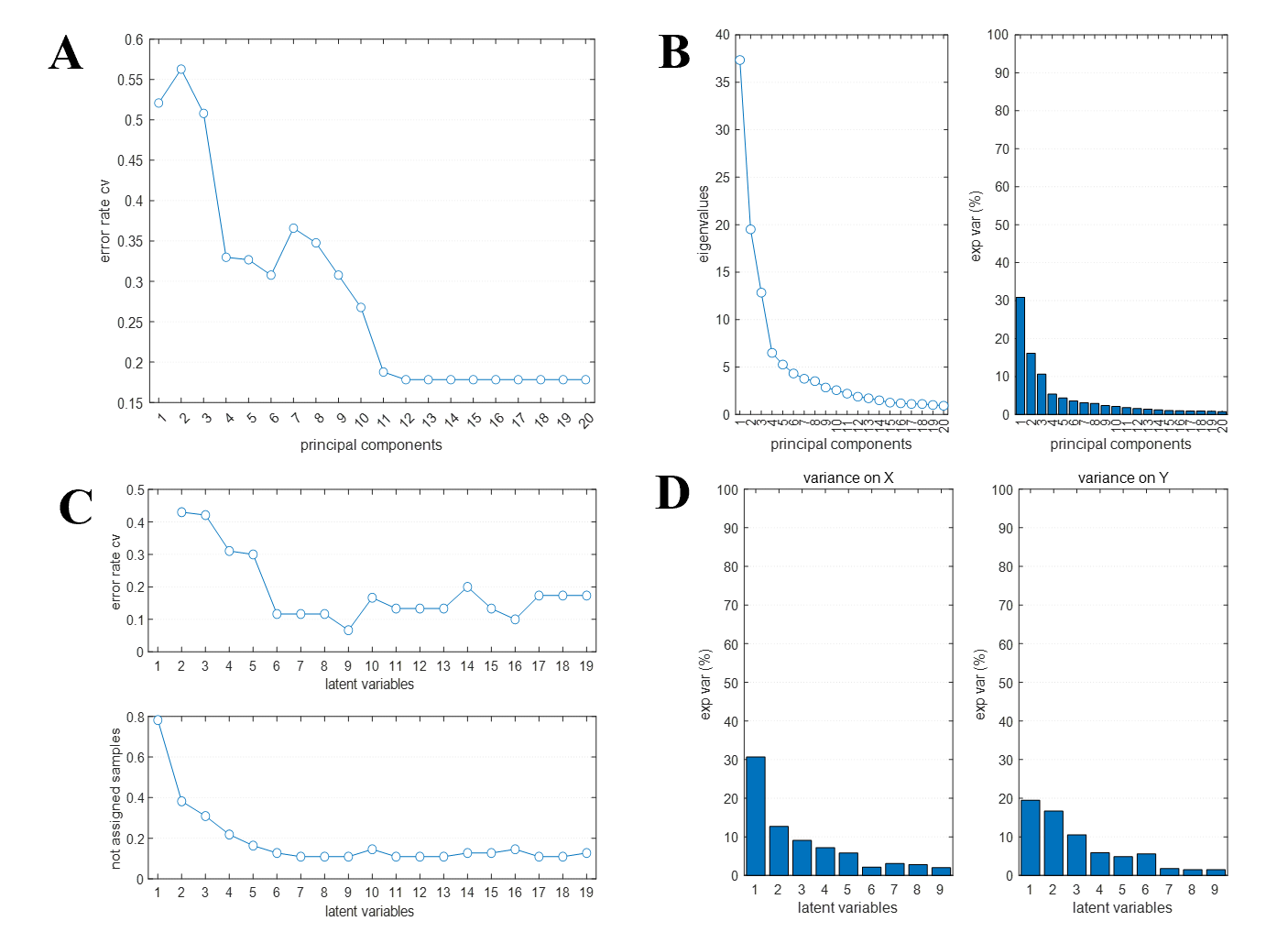


**Figure 7.** The cross-validation error map and explained variation map of the *Amomi fructus* origin identification model based on data fusion. **(A)** Principal component fraction-cross validation error plot in PCA-DA model. **(B)** Variation infographic of PCA-DA latent variable interpretation. **(C)** The number of latent variables-cross validation error plot in PLS-DA model. **(D)** Variation infographic of PLS-DA latent variable interpretation.


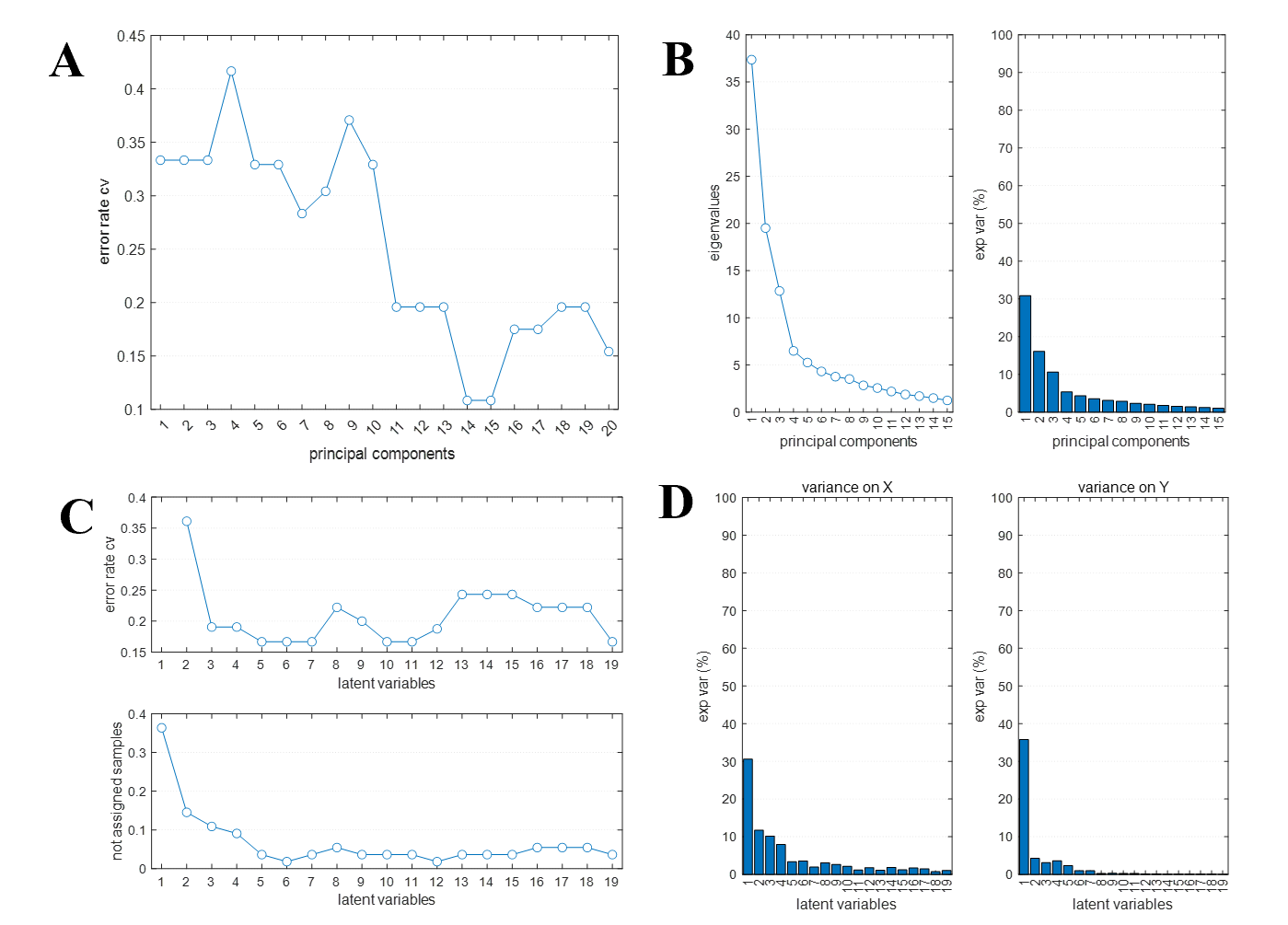


**Figure 8.** The cross validation error map and the explained variation map of the provenance identification model of *Amomi fructus* based on data fusion are established. **(A)** Principal component fraction-cross validation error plot in PCA-DA model. **(B)** Variation infographic of PCA-DA latent variable interpretation. **(C)** The number of latent variables-cross validation error plot in PLS-DA model. **(D)** Variation infographic of PLS-DA latent variable interpretation.

## Supplementary Tables

**Table 1.** Batch information of *Amomi fructus*

| No. | Origin information | Manufacturer |
| --- | --- | --- |
| S1 | Yunnan Province | Bozhou Zhangzhongjing Traditional Chinese Medicine Co., Ltd. |
| S2 | Yunnan Province | Henan Hongbo Pharmaceutical Co., Ltd. |
| S3 | Yunnan Province | Henan Hongbo Pharmaceutical Co., Ltd. |
| S4 | Yunnan Province | Bozhou Zhangzhongjing Traditional Chinese Medicine Co., Ltd. |
| S5 | Yunnan Province | Bozhou Zhangzhongjing Traditional Chinese Medicine Co., Ltd. |
| S6 | Yunnan Province | Bozhou Zhangzhongjing Traditional Chinese Medicine Co., Ltd. |
| S7 | Yunnan Province | Yunnan Kesi Pharmaceutical Co., Ltd. |
| S8 | Yunnan Province | Bozhou Zhangzhongjing Traditional Chinese Medicine Co., Ltd. |
| S9 | Yunnan Province | Yunnan Kesi Pharmaceutical Co., Ltd. |
| S10 | Yunnan Province | Bozhou Zhangzhongjing Traditional Chinese Medicine Co., Ltd. |
| S11 | Yunnan Province | Bozhou Shenglin Pharmaceutical Co., Ltd. |
| S12 | Yunnan Province | Hebei Kang yi qiang Pharmaceutical Co., Ltd. |
| S13 | Yunnan Province | Hebei Rongshun Pharmaceutical Co., Ltd. |
| S14 | Yunnan Province | Hebei Xinjingyuan Pharmaceutical Co., Ltd. |
| S15 | Yunnan Province | Hebei Renxin Pharmaceutical Co., Ltd. |
| S16 | Yunnan Province | Anhui Shenghaitang Traditional Chinese Medicine Co., Ltd. |
| S17 | Yunnan Province | Hunan Chunkehui Traditional Chinese Medicine Co., Ltd. |
| S18 | Yunnan Province | Hebei Qixin Chinese Medicine Granules Co., Ltd. |
| S19 | Yunnan Province | Hunan Songlingtang Traditional Chinese Medicine Co., Ltd. |
| S20 | Yunnan Province | Guizhou Xianghetang Traditional Chinese Medicine Co., Ltd. |
| S21 | Yunnan Province | Guangdong Hebaicao Pharmaceutical Co., Ltd. |
| S22 | Guangdong province | Zhejiang Tongjuntang Traditional Chinese Medicine Co., Ltd. |
| S23 | Guangdong province | Zhengzhou Ruilong Pharmaceutical Co., Ltd. |
| S24 | Guangdong province | Henan Qianfang Pharmaceutical Co., Ltd. |
| S25 | Guangdong province | Hunan Honghua Traditional Chinese Medicine Co., Ltd. |
| S26 | Guangdong province | Jinan Hebao Chinese Herbal Medicine Co., Ltd. |
| S27 | Guangdong province | Anhui Huisongtang Guoji Pharmaceutical Co., Ltd. |
| S28 | Guangdong province | Yongzhou Yongdu Traditional Chinese Medicine Co., Ltd. |
| S29 | Guangdong province | Hunan Juren Traditional Chinese Medicine Co., Ltd. |
| S30 | Guangdong province | Hunan Ranruntang Traditional Chinese Medicine Co., Ltd. |
| S31 | Guangdong province | Shaoyang Shennong Traditional Chinese Medicine Technology Development Co., Ltd. |
| S32 | Guangdong province | Jiangxi Jiuzhoutang Traditional Chinese Medicine Co., Ltd. |
| S33 | Guangdong province | Chongqing Houjie Pharmaceutical Co., Ltd. |
| S34 | Guangdong province | Jiangyou City Forest Planting Professional Cooperatives |
| S35 | Guangxi province | Chengdu Lotus Pond Medicinal Materials Professional Market |
| S36 | Guangxi province | Guangxi Yulin Chinese herbal medicine professional market |
| S37 | Guangxi province | Chengdu Lotus Pond Medicinal Materials Professional Market |
| S38 | Guangxi province | Chengdu Lotus Pond Medicinal Materials Professional Market |
| S39 | Yunnan Province | Jiangyou City Forest Planting Professional Cooperatives |
| S40 | Yunnan Province | Chengdu Lotus Pond Medicinal Materials Professional Market |
| S41 | Unknown | Guangxi Yulin Chinese herbal medicine professional market |
| S42 | Unknown | Chengdu Lotus Pond Medicinal Materials Professional Market |
| S43 | Unknown | Chengdu Lotus Pond Medicinal Materials Professional Market |
| S44 | Guangxi province | Hebei Wandong Pharmaceutical Co., Ltd |
| S45 | Yunnan Province | Guangdong Yiyun Pharmaceutical Co., Ltd. |
| S46 | Hainan Province | Anxing Traditional Chinese Medicine Co., Ltd. |
| S47 | Hainan Province | Guangdong Qingping Chinese herbal medicine wholesale market |
| S48 | Hainan Province | Chengdu Lotus Pond Medicinal Materials Professional Market |
| S49 | Hainan Province | Bozhou Chinese herbal medicine wholesale market |
| S50 | Hainan Province | Sichuan Zangxitang Biological Technology Co., Ltd. |
| S51 | Myanmar | Chengdu Jinniu District Highland Herbs Food Business Department |
| S52 | Myanmar | Chengdu Jinniu District Highland Herbs Food Business Department |
| S53 | Myanmar | Chengdu Jinniu District Highland Herbs Food Business Department |
| S54 | Myanmar | Chengdu Lotus Pond Medicinal Materials Professional Market |
| S55 | Myanmar | Chengdu Lotus Pond Medicinal Materials Professional Market |
| S56 | Hainan Province | Hongya County Wawushan Pharmaceutical Co., Ltd. |
| S57 | Guangxi province | Jiangxi Heshuo Pharmaceutical Co., Ltd. |
| S58 | Guangxi province | Hebei Wandong Pharmaceutical Co., Ltd. |
| S59 | Guangxi province | Huazhou Huayi Traditional Chinese Medicine Co., Ltd. |
| S60 | Guangxi province | Hunan Yafei Traditional Chinese Medicine Co., Ltd. |
| S61 | Guangxi province | Hubei Jingui Traditional Chinese Medicine Co., Ltd. |
| S62 | Guangxi province | Hunan Ranruntang Traditional Chinese Medicine Co., Ltd. |
| S63 | Guangxi province | Chongqing Zhongmiao Pharmaceutical Co., Ltd. |
| S64 | Guangxi province | Chongqing Guochengtang Pharmaceutical Co., Ltd. |
| S65 | Guangxi province | Hebei Kangyiqiang Pharmaceutical Co., Ltd. |
| S66 | Guangxi province | Jiangxi Bairen Traditional Chinese Medicine Co., Ltd. |
| S67 | Guangxi province | Shaoyang Shennong Traditional Chinese Medicine Technology Development Co., Ltd. |
| S68 | Guangxi province | Jiangxi Jiuzhoutang Traditional Chinese Medicine Co., Ltd. |
| S69 | Hainan Province | Anhui Shenghaitang Traditional Chinese Medicine Co., Ltd. |
| S70 | Guangxi province | Jiangxi Zhihetang Traditional Chinese Medicine Co., Ltd. |
| S71 | Guangxi province | Sichuan Gukang Pharmaceutical Co., Ltd. |
| S72 | Guangdong province | Hubei Jingui Traditional Chinese Medicine Co., Ltd. |
| S73 | Guangxi province | Sichuan Tuojiangyuan Pharmaceutical Co., Ltd. |
| S74 | Guangxi province | Hebei Guosongtang Pharmaceutical Co., Ltd. |
| S75 | Hainan Province | Hebei Renxin Pharmaceutical Co., Ltd. |

**Table 2.** Sensors information

| No. | Sensor name | Sensor description and sensitivities |
| --- | --- | --- |
| 1 | W1C | Aromatic components (benzenes) |
| 2 | W5S | Nitrogen oxide |
| 3 | W3C | Aromatic components (bmines) |
| 4 | W6S | Hydride |
| 5 | W5C | Aromatic components of alkanes (short-chain alkanes) |
| 6 | W1S | Methane (methyl) |
| 7 | W1W | Sulfide (inorganic sulfide ) |
| 8 | W2S | Ethanol (alcohols) |
| 9 | W2W | Sulfides (organic sulfides) |
| 10 | W3S | Long-chain alkanes |

**Table 3.** Compound list information

| No. | Type | Compound | CAS | Formula | MW | RI | Rt/s | Dt/ms |
| --- | --- | --- | --- | --- | --- | --- | --- | --- |
| 1 | Esters | 5-methylquinoxaline | C13708128 | C_9_H_8_N_2_ | 144.2 | 1489.5 | 1116.552 | 1.22184 |
| 2 |  | (Z)-3-Hexenyl hexanoate | C31501118 | C_12_H_22_O_2_ | 198.3 | 1422.6 | 865.026 | 2.18858 |
| 3 |  | Linalyl butyrate | C78364 | C_14_H_24_O_2_ | 224.3 | 1413.4 | 835.05 | 2.02673 |
| 4 |  | 2-Phenylethyl butanoate-M | C103526 | C_12_H_16_O_2_ | 192.3 | 1445.2 | 942.727 | 1.43242 |
| 5 |  | 2-Phenylethyl butanoate-D | C103526 | C_12_H_16_O_2_ | 192.3 | 1445.5 | 943.793 | 1.47409 |
| 6 |  | Butanoic acid, 3-methyl-, 2-phenylethyl ester | C140261 | C_13_H_18_O_2_ | 206.3 | 1501.3 | 1167.801 | 1.47723 |
| 7 |  | δ-Nonalactone | C3301948 | C_9_H_16_O_2_ | 156.2 | 1389.2 | 761.402 | 1.85793 |
| 8 |  | Linalyl isobutyrate | C78353 | C_14_H_24_O_2_ | 224.3 | 1368.8 | 704.286 | 1.21874 |
| 9 |  | Methyl-2-methoxy-benzoate | C606451 | C_9_H_10_O_3_ | 166.2 | 1347.3 | 648.919 | 1.22674 |
| 10 |  | Methyl anisate | C121982 | C_9_H_10_O_3_ | 166.2 | 1328 | 602.975 | 1.79578 |
| 11 |  | Methyl 2-nonynoate | C111808 | C_10_H_16_O_2_ | 168.2 | 1315.1 | 573.801 | 1.46161 |
| 12 |  | Allyl (3-methylbutoxy)acetate | C67634008 | C_10_H_18_O_3_ | 186.3 | 1247 | 442.619 | 1.40785 |
| 13 |  | Ethyl 2-hydroxybenzoate-M | C118616 | C_9_H_10_O_3_ | 166.2 | 1259.3 | 463.783 | 1.26461 |
| 14 |  | Ethyl 2-hydroxybenzoate-D | C118616 | C_9_H_10_O_3_ | 166.2 | 1259.1 | 463.583 | 1.30926 |
| 15 |  | Linalool acetate | C115957 | C_12_H_20_O_2_ | 196.3 | 1240.4 | 431.668 | 1.21743 |
| 16 |  | Citronellyl acetate | C150845 | C_12_H_22_O_2_ | 198.3 | 1332.9 | 614.307 | 1.47797 |
| 17 |  | Bornyl acetate | C76493 | C_12_H_20_O_2_ | 196.3 | 1279.7 | 501.418 | 1.22576 |
| 18 |  | 6-Octen-1-ol, 3,7-dimethyl-, formate | C105851 | C_11_H_20_O_2_ | 184.3 | 1269.9 | 483.043 | 1.35253 |
| 19 |  | 5-Propyldihydro-2(3H)-furanone | C105215 | C_7_H_12_O_2_ | 128.2 | 1170.2 | 335.744 | 1.65693 |
| 20 |  | propionic acid hexyl ester | C2445763 | C_9_H_18_O_2_ | 158.2 | 1113.9 | 274.873 | 1.44614 |
| 21 |  | (Z)-3-Hexenyl butyrate | C16491364 | C_10_H_18_O_2_ | 170.3 | 1221.7 | 403.71 | 1.42955 |
| 22 |  | Methyl 2-hydroxybenzoate | C119368 | C_8_H_8_O_3_ | 152.1 | 1184.8 | 353.694 | 1.16172 |
| 23 |  | Butyl hexanoate | C626824 | C_10_H_20_O_2_ | 172.3 | 1182.8 | 351.186 | 1.46182 |
| 24 |  | 2-Hydroxy-benzoic acid methyl ester | C119368 | C_8_H_8_O_3_ | 152.1 | 1166 | 330.704 | 1.15602 |
| 25 |  | Allyl heptanoate | C142198 | C_10_H_18_O_2_ | 170.3 | 1163.9 | 328.196 | 1.44853 |
| 26 |  | Benzyl acetate | C140114 | C_9_H_10_O_2_ | 150.2 | 1103.4 | 264.88 | 1.32103 |
| 27 |  | benzoic acid ethyl ester | C93890 | C_9_H_10_O_2_ | 150.2 | 1169.6 | 335.005 | 1.278 |
| 28 |  | Butanoic acid, butyl ester | C109217 | C_8_H_16_O_2_ | 144.2 | 984 | 180.798 | 1.33698 |
| 29 |  | Benzenepropanoic acid, ethyl ester | C2021285 | C_11_H_14_O_2_ | 178.2 | 1345 | 643.207 | 1.87135 |
| 30 |  | Allyl phenoxyacetate-M | C7493745 | C_11_H_12_O_3_ | 192.2 | 1483 | 1088.96 | 1.86897 |
| 31 |  | Allyl phenoxyacetate-D | C7493745 | C_11_H_12_O_3_ | 192.2 | 1482.3 | 1086.079 | 1.88988 |
| 32 |  | 2-Butenoic acid,ethyl ester,(E)- | C623701 | C_6_H_10_O_2_ | 114.1 | 829.5 | 130.422 | 1.19364 |
| 33 | Ketones | β-Ionone-M | C79776 | C_13_H_20_O | 192.3 | 1479.4 | 1074.331 | 1.46516 |
| 34 |  | β-Ionone-D | C79776 | C_13_H_20_O | 192.3 | 1479.3 | 1073.798 | 1.48748 |
| 35 |  | p-Methoxyacetophenone | C100061 | C_9_H_10_O_2_ | 150.2 | 1363.6 | 690.617 | 1.24348 |
| 36 |  | Damascenone | C23696857 | C_13_H_18_O | 190.3 | 1343.9 | 640.613 | 1.38562 |
| 37 |  | 2-Cyclohexen-1-one, 2-methyl-5-(1-methylethenyl)- | C99490 | C_10_H_14_O | 150.2 | 1246.1 | 440.993 | 1.82282 |
| 38 |  | 3,5-Heptadien-2-one, 6-methyl- | C1604280 | C_8_H_12_O | 124.2 | 1102.6 | 264.101 | 1.74583 |
| 39 |  | 4-Ketoisophorone | C1125219 | C_9_H_12_O_2_ | 152.2 | 1157.1 | 320.254 | 1.33646 |
| 40 |  | 1-Methyl-2-pyrrolidone | C872504 | C_5_H_9_NO | 99.1 | 1092.8 | 255.08 | 1.44442 |
| 41 |  | 2,3-Dihydro-5-methylfuran-2-one | C591128 | C_5_H_6_O_2_ | 98.1 | 924.8 | 161.167 | 1.37707 |
| 42 |  | Cyclohexanone | C108941 | C_6_H_10_O | 98.1 | 832.1 | 131.227 | 1.15478 |
| 43 |  | 2-Hexanone | C591786 | C_6_H_12_O | 100.2 | 797 | 120.903 | 1.19479 |
| 44 |  | (Z)-Jasmone | C488108 | C_11_H_16_O | 164.2 | 1435.8 | 909.475 | 1.32207 |
| 45 |  | 1-(acetyloxy)-2-propanone | C592201 | C_5_H_8_O_3_ | 116.1 | 824.9 | 129.041 | 1.11614 |
| 46 | Alcohols | (E)-geraniol-M | C106241 | C_10_H_18_O | 154.3 | 1268.2 | 479.839 | 1.6937 |
| 47 |  | (E)-geraniol-D | C106241 | C_10_H_18_O | 154.3 | 1268.2 | 479.839 | 1.72398 |
| 48 |  | β-Phenyl ethyl alcohol | C60128 | C_8_H_10_O | 122.2 | 1090.7 | 253.222 | 1.296 |
| 49 |  | 2-Furanmethanol, 5-ethenyltetrahydro-α,α,5-trimethyl- | C60047178 | C_10_H_18_O_2_ | 170.3 | 1073.3 | 238.094 | 1.26312 |
| 50 |  | 3-Octen-1-ol, (Z)- | C20125842 | C_8_H_16_O | 128.2 | 1068.8 | 234.391 | 1.36101 |
| 51 |  | 1-Octanol-M | C111875 | C_8_H_18_O | 130.2 | 1071 | 236.134 | 1.472 |
| 52 |  | 1-Octanol-D | C111875 | C_8_H_18_O | 130.2 | 1073.1 | 237.876 | 1.55954 |
| 53 |  | 5-methyl-2-Furanmethanol | C3857258 | C_6_H_8_O_2_ | 112.1 | 1005.3 | 193.014 | 1.26864 |
| 54 |  | Rhodinol | C6812788 | C_10_H_20_O | 156.3 | 1243 | 435.886 | 1.33069 |
| 55 |  | Anisyl alcohol | C105135 | C_8_H_10_O_2_ | 138.2 | 1262.4 | 469.399 | 1.07922 |
| 56 |  | Nerol | C106252 | C_10_H_18_O | 154.3 | 1256.1 | 458.276 | 1.23038 |
| 57 |  | 3-Heptanol | C589822 | C_7_H_16_O | 116.2 | 872.7 | 144.228 | 1.3364 |
| 58 | Alkenes | (E)-Caryophyllene-M | C87445 | C_15_H_24_ | 204.4 | 1420.4 | 857.747 | 1.43314 |
| 59 |  | (E)-Caryophyllene-D | C87445 | C_15_H_24_ | 204.4 | 1419.8 | 855.821 | 1.52132 |
| 60 |  | (Z)- β-Farnesene | C28973979 | C_15_H_24_ | 204.4 | 1453.4 | 972.622 | 1.48807 |
| 61 |  | β-Elemene | C515139 | C_15_H_24_ | 204.4 | 1361.3 | 684.584 | 1.44884 |
| 62 |  | Longifolene-M | C475207 | C_15_H_24_ | 204.4 | 1378.1 | 729.851 | 1.53291 |
| 63 |  | Longifolene-D | C475207 | C_15_H_24_ | 204.4 | 1377.2 | 727.381 | 2.14557 |
| 64 |  | D-Limonene | C138863 | C_10_H_16_ | 136.2 | 1025.5 | 205.304 | 1.29629 |
| 65 |  | α-Phellanderene | C99832 | C_10_H_16_ | 136.2 | 1009 | 195.192 | 1.23348 |
| 66 |  | α-Terpinolene | C586629 | C_10_H_16_ | 136.2 | 1108.4 | 269.516 | 1.72266 |
| 67 |  | β -Pinene | C127913 | C_10_H_16_ | 136.2 | 979.5 | 179.234 | 1.22146 |
| 68 |  | α- pinene | C80568 | C_10_H_16_ | 136.2 | 901 | 153.893 | 1.22219 |
| 69 | Phenols | 4-ethyl-2-methoxyphenol | C2785899 | C_9_H_12_O_2_ | 152.2 | 1350.7 | 657.432 | 1.25326 |
| 70 |  | p-propylguaiacol | C2785877 | C_10_H_14_O_2_ | 166.2 | 1329.1 | 605.278 | 1.31062 |
| 71 |  | Sesamol | C533313 | C_7_H_6_O_3_ | 138.1 | 1298.9 | 539.486 | 1.20146 |
| 72 |  | meta-Cresol | C108394 | C_7_H_8_O | 108.1 | 1284.3 | 510.277 | 1.10814 |
| 73 |  | 2-Methoxyphenol | C90051 | C_7_H_8_O_2_ | 124.1 | 1107.4 | 268.59 | 1.11647 |
| 74 |  | 4-Methylguaiacol-M | C93516 | C_8_H_10_O_2_ | 138.2 | 1227 | 411.504 | 1.18519 |
| 75 |  | 4-Methylguaiacol-D | C93516 | C_8_H_10_O_2_ | 138.2 | 1227.5 | 412.177 | 1.21134 |
| 76 | Pyrazines | 2-acetoxy-3,5-dichloro-benzonitrile | C54300082 | C_9_H_5_Cl_2_NO_2_ | 230 | 1227.5 | 412.153 | 1.27632 |
| 77 |  | Cumin aldehyde | C122032 | C_10_H_12_O | 148.2 | 1229.7 | 415.479 | 1.89599 |
| 78 |  | 3- sec-butyl-2-methoxypyrazine | C24168705 | C_9_H_14_N_2_O | 166.2 | 1182.2 | 350.451 | 1.72506 |
| 79 |  | 2-ethyl-3,5-dimethyl pyrazine-M | C13925070 | C_8_H_12_N_2_ | 136.2 | 1060.6 | 228.643 | 1.73221 |
| 80 | Phenols | 2-ethyl-3,5-dimethyl pyrazine-D | C13925070 | C_8_H_12_N_2_ | 136.2 | 1060 | 228.195 | 1.75537 |
| 81 |  | 2-ethyl-5-methyl pyrazine-M | C13360640 | C_7_H_10_N_2_ | 122.2 | 1019.7 | 201.676 | 1.67065 |
| 82 |  | 2-ethyl-5-methyl pyrazine-D | C13360640 | C_7_H_10_N_2_ | 122.2 | 1021.5 | 202.825 | 1.73309 |
| 83 | Aldehydes | 3,4-Dimethoxybenzaldehyde | C120149 | C_9_H_10_O_3_ | 166.2 | 1402.1 | 799.731 | 1.34341 |
| 84 |  | 2-Phenyl-2-butenal | C4411896 | C_10_H_10_O | 146.2 | 1311.2 | 565.359 | 1.25458 |
| 85 |  | anisaldehyde | C123115 | C_8_H_8_O_2_ | 136.1 | 1347 | 648.215 | 1.67944 |
| 86 |  | Citral | C5392405 | C_10_H_16_O | 152.2 | 1243.7 | 437.017 | 1.06507 |
| 87 | Ethers | Diphenyl oxide | C101848 | C_12_H_10_O | 170.2 | 1277.1 | 496.527 | 1.29521 |
| 88 |  | Veratrole | C91167 | C_8_H_10_O_2_ | 138.2 | 1170.2 | 335.744 | 1.59931 |
| 89 |  | 2-furfuryl methyl disulfide | C57500002 | C_6_H_8_OS_2_ | 160.2 | 1208.5 | 385.107 | 1.21624 |
| 90 | Acids | 1-Butanol, 3-methyl-, benzoate | C94462 | C_12_H_16_O_2_ | 192.3 | 1420 | 856.352 | 1.47022 |
| 91 |  | 2-Methylbutanoic acid | C116530 | C_5_H_10_O_2_ | 102.1 | 802.4 | 122.451 | 1.47488 |
| 92 | Others | Benzothiazole | C95169 | C_7_H_5_NS | 135.2 | 1284 | 509.731 | 1.17398 |
| 93 |  | 2-Formyl-5-methylthiophene | C13679704 | C_6_H_6_OS | 126.2 | 1068.5 | 234.174 | 1.17213 |
| 94 |  | 2-Acetylthiazole | C24295032 | C_5_H_5_NOS | 127.2 | 1004.2 | 192.361 | 1.48027 |
| 95 |  | Geosmin | C19700211 | C_12_H_22_O | 182.3 | 1437.1 | 914.076 | 1.36666 |
| 96 |  | Hydrocoumarin | C119846 | C_9_H_8_O_2_ | 148.2 | 1361.5 | 685.027 | 1.72987 |
| 97 |  | 1,2-dimethoxy-4-allylbenzene | C93152 | C_11_H_14_O_2_ | 178.2 | 1375.7 | 723.074 | 1.4522 |
| 98 |  | Anethol | C104461 | C_10_H_12_O | 148.2 | 1336.7 | 623.171 | 1.18603 |
| 99 |  | 2-methylnaphthalene | C91576 | C_11_H_10_ | 142.2 | 1238.8 | 429.197 | 1.18916 |
| 100 |  | Heptane, 2,2,4,6,6-pentamethyl-M | C13475826 | C_12_H_26_ | 170.3 | 982.5 | 180.266 | 1.37373 |
| 101 | Acids | Heptane, 2,2,4,6,6-pentamethyl-D | C13475826 | C_12_H_26_ | 170.3 | 982.5 | 180.266 | 1.7294 |
| 102 | Unidentified | Unknown1 | - | - | - | 1417 | 846.596 | 2.44027 |
| 103 |  | Unknown2 | - | - | - | 1487.2 | 1106.833 | 1.29997 |
| 104 |  | Unknown3 | - | - | - | 1579.8 | 1575.445 | 1.26212 |
| 105 |  | Unknown4 | - | - | - | 1372.6 | 714.807 | 1.15301 |
| 106 |  | Unknown5 | - | - | - | 1232.7 | 419.88 | 1.38764 |
| 107 |  | Unknown6 | - | - | - | 1355.6 | 669.764 | 1.1689 |
| 108 |  | Unknown7 | - | - | - | 1204.7 | 379.86 | 1.07833 |
| 109 |  | Unknown8 | - | - | - | 1380.1 | 735.518 | 1.26198 |
| 110 |  | Unknown9 | - | - | - | 1394 | 775.602 | 1.15175 |
| 111 |  | Unknown10 | - | - | - | 1343.5 | 639.607 | 1.80342 |

*CAS#, Chemical Abstracts Service registry number.

MW, molecular weight.

RI, retention index calculated using n-ketones C4-C9 as external standard.

RT, retention time in the capillary GC column.

DT, drift time in the drift tube.

**Table 4.** The selected VIP> 1, and P < 0.05 compound information

| No. | Var ID | VIP | Probability (AO) | Probability (AK) |
| --- | --- | --- | --- | --- |
| 1 | δ-Nonalactone | 1.31159 | 0.00000 | 0.00000 |
| 2 | Unknown7 | 1.30362 | 0.00000 | 0.00460 |
| 3 | 2-Acetylthiazole | 1.29655 | 0.00000 | 0.00957 |
| 4 | β-Ionone-M | 1.29568 | 0.00000 | 0.00149 |
| 5 | β--Ionone-D | 1.29342 | 0.00000 | 0.00393 |
| 6 | (Z)- β-Farnesene | 1.28956 | 0.00000 | 0.00000 |
| 7 | (E)-geraniol-M | 1.28938 | 0.00031 | 0.00000 |
| 8 | 2,3-Dihydro-5-methylfuran-2-one | 1.28729 | 0.00000 | 0.00033 |
| 9 | 2-Phenylethyl butanoate-D | 1.27904 | 0.00000 | 0.00000 |
| 10 | 1-Methyl-2-pyrrolidone | 1.27693 | 0.00000 | 0.00084 |
| 11 | 5-Propyldihydro-2(3H)-furanone | 1.27063 | 0.00000 | 0.00000 |
| 12 | 3- sec-butyl-2-methoxypyrazine | 1.26697 | 0.00000 | 0.00000 |
| 13 | 2-Furanmethanol, 5-methyl- | 1.26516 | 0.04116 | 0.00000 |
| 14 | 5-methylquinoxaline | 1.26222 | 0.00000 | 0.00000 |
| 15 | 1-Octanol-M | 1.25759 | 0.00000 | 0.00000 |
| 16 | D-Limonene | 1.25019 | 0.00000 | 0.01427 |
| 17 | Unknown2 | 1.24561 | 0.00000 | 0.00000 |
| 18 | 3,4-Dimethoxybenzaldehyde | 1.24465 | 0.00000 | 0.00000 |
| 19 | α- pinene | 1.24178 | 0.00000 | 0.00065 |
| 20 | Allyl heptanoate | 1.23964 | 0.00000 | 0.00000 |
| 21 | Linalool acetate | 1.23679 | 0.00000 | 0.00000 |
| 22 | 2-Hexanone | 1.2294 | 0.00000 | 0.02872 |
| 23 | p-propylguaiacol | 1.22898 | 0.00000 | 0.00000 |
| 24 | 2-ethyl-3,5-dimethyl pyrazine-M | 1.21899 | 0.00000 | 0.00000 |
| 25 | (E)-Caryophyllene-D | 1.21001 | 0.00009 | 0.00000 |
| 26 | 2-Furanmethanol,5-ethenyltetrahydro-α,α,5-trimethyl- | 1.20571 | 0.00000 | 0.00000 |
| 27 | 2-Formyl-5-methylthiophene | 1.20306 | 0.00000 | 0.00000 |
| 28 | Heptane, 2,2,4,6,6-pentamethyl-M | 1.1892 | 0.00000 | 0.00719 |
| 29 | Linalyl isobutyrate | 1.18808 | 0.00000 | 0.00000 |
| 30 | 3-Octen-1-ol, (Z)- | 1.18612 | 0.00000 | 0.00000 |
| 31 | 2-ethyl-5-methyl pyrazine-D | 1.1809 | 0.00000 | 0.00125 |
| 32 | Unknown9 | 1.17511 | 0.00000 | 0.00000 |
| 33 | Diphenyl oxide | 1.17478 | 0.00000 | 0.01750 |
| 34 | 2-Phenylethyl butanoate-M | 1.16767 | 0.00000 | 0.00010 |
| 35 | 2-ethyl-5-methyl pyrazine-M | 1.15439 | 0.00000 | 0.00266 |
| 36 | Methyl-2-methoxy-benzoate | 1.13519 | 0.00000 | 0.00007 |
| 37 | α-Terpinolene | 1.13508 | 0.00470 | 0.00000 |
| 38 | Allyl phenoxyacetate-M | 1.12143 | 0.00000 | 0.00000 |
| 39 | β-Elemene | 1.11918 | 0.00000 | 0.00004 |
| 40 | Unknown6 | 1.09349 | 0.00164 | 0.00000 |
| 41 | (Z)-3-Hexenyl hexanoate | 1.08122 | 0.00000 | 0.00000 |
| 42 | Bornyl acetate | 1.07978 | 0.00000 | 0.00000 |
| 43 | 1-Octanol-D | 1.07912 | 0.00000 | 0.00000 |
| 44 | 2-ethyl-3,5-dimethyl pyrazine-D | 1.07428 | 0.00000 | 0.00000 |
| 45 | 1-Butanol, 3-methyl-, benzoate | 1.03971 | 0.00000 | 0.00000 |
| 46 | Allyl phenoxyacetate-D | 1.02124 | 0.00000 | 0.00000 |
| 47 | 2-Methoxyphenol | 1.01701 | 0.00000 | 0.00000 |
